# Tracing population movements in ancient East Asia through the linguistics and archaeology of textile production

**DOI:** 10.1017/ehs.2020.4

**Published:** 2020-02-14

**Authors:** Sarah Nelson, Irina Zhushchikhovskaya, Tao Li, Mark Hudson, Martine Robbeets

**Affiliations:** 1 Department of Anthropology, University of Denver, Denver, CO, USA; 2 Laboratory of Medieval Archaeology, Institute of History, Archaeology and Ethnography of Peoples of Far East, Far Eastern Branch of Russian Academy of Sciences, Vladivostok, Russia; 3 Eurasia3angle Research group, Max Planck Institute for the Science of Human History, Jena, Germany; 4 Department of Archaeology, Wuhan University, Wuhan, China

**Keywords:** Archaeolinguistics, Language/Farming Dispersal Hypothesis, Transeurasian language family, textile technology, Northeast Asia

## Abstract

Archaeolinguistics, a field which combines language reconstruction and archaeology as a source of information on human prehistory, has much to offer to deepen our understanding of the Neolithic and Bronze Age in Northeast Asia. So far, integrated comparative analyses of words and tools for textile production are completely lacking for the Northeast Asian Neolithic and Bronze Age. To remedy this situation, here we integrate linguistic and archaeological evidence of textile production, with the aim of shedding light on ancient population movements in Northeast China, the Russian Far East, Korea and Japan. We show that the transition to more sophisticated textile technology in these regions can be associated not only with the adoption of millet agriculture but also with the spread of the languages of the so-called ‘Transeurasian’ family. In this way, our research provides indirect support for the Language/Farming Dispersal Hypothesis, which posits that language expansion from the Neolithic onwards was often associated with agricultural colonization.

## Graphical abstract

**Figure:**
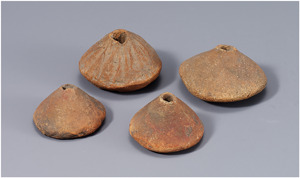

**Media summary:** Archaeolinguistics, which combines language reconstruction and archaeology as a source of information on human prehistory, has much to offer to deepen our understanding of the Neolithic and Bronze Age in Northeast Asia. Here we integrate the language and archaeology of textile production as a marker of agricultural dispersal and population migration in Northeast China, the Russian Far East, Korea and Japan. We show that the transition to more sophisticated textile technology in these regions can be associated with the adoption of millet agriculture and the spread of the so-called ‘Transeurasian’ language family. In this way, our research provides indirect support for the Language/Farming Dispersal Hypothesis, which posits that language expansion from the Neolithic onwards was often associated with agricultural colonization.

## Introduction

The Language/Farming Dispersal Hypothesis makes the radical and controversial claim that many of the world's major language families owe their premodern distribution to the adoption of agriculture by their early speakers (Bellwood 1984; Renfrew 1987; Bellwood and Renfrew 2002; Diamond and Bellwood 2003; Bellwood 2005, 2011; Robbeets and Savelyev 2017). The standard technique to test this hypothesis for a given language family is to use reconstructed words with an agricultural meaning as a way of determining whether the ancestral speakers were familiar with agriculture. This method has been used to support or reject agriculture-driven language spread for a wide range of language families such as Indo-European (Comrie 2002; Anthony 2007; Kroonen 2012; Iversen and Kroonen 2017; Kümmel 2017), Sino-Tibetan (Sagart *et al.* 2019), Austronesian (Blust 1995, 2013; Pawley 2002), Austroasiatic (Sidwell and Blench 2011; van Driem 2017), Trans-New Guinean (Schapper 2017), Bantu (Philipson 2002; Bostoen and Koni Muluwa 2017), Arawak (Aikhenvald 1999) and Otomanguean (Kaufman 1990; Brown 2015). Recently, agricultural vocabulary has also been attributed to the ancestor of the Transeurasian languages, supporting the claim that the primary dispersal of these languages was agriculture-driven (Robbeets 2017, 2020a).

**Figure 1.**
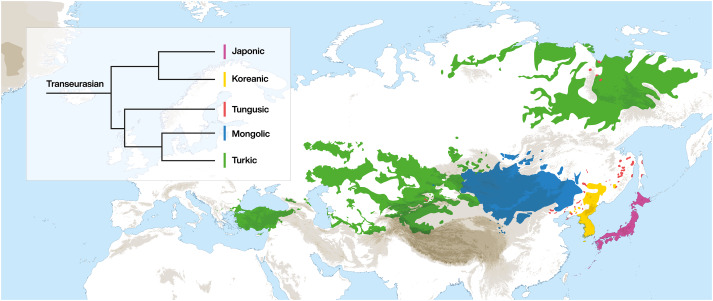
The distribution and classification of the Transeurasian languages.

The term ‘Transeurasian’ replaces the traditional label ‘Altaic’ and refers to the large group of geographically adjacent languages, stretching across Europe and Asia, presented in Figure 1. It includes five uncontroversial linguistic families: Japonic, Koreanic, Tungusic, Mongolic and Turkic. The question of whether these five groups descend from a single common ancestor has been the topic of a longstanding debate (Starostin *et al.* 2003; Vovin 2005; Dybo and Starostin 2008; Johanson 2010). Recent assessments have shown that even if the historical relationship between the Transeurasian languages is heavily marked by borrowing, there is nonetheless a core of reliable evidence for the classification of Transeurasian as a valid genealogical grouping (Robbeets 2005, 2015, 2020b, c).

Here we will test the claim that Transeurasian language dispersal was initially agriculture-driven by investigating words and material evidence beyond agriculture. The logic behind this approach is that when agriculture drives people to move along with the language they speak, we expect that, besides crops and farming technology, other aspects of material culture will move as well. Given the importance of textile production in the ancient cultures and languages of the Transeurasian-speaking region, we here search for a correlation between the use of agriculture, textile production and the words reflecting these activities. To this end, we will first compare words for textile production across the Transeurasian languages and reconstruct them back to the common ancestral language. These reconstructions will enable us to make predictions about the prehistory of textile production in the Transeurasian-speaking region. We will verify our predictions through a comparative survey of textile technology in Northeast China, the Russian Far East, Korea and Japan in the Neolithic and Bronze Age. Finally, we will compare the distribution of tools and words and map our linguistic inferences on those from archaeology.

While studies on the linguistics of textile production in individual Transeurasian languages are extremely rare (for Japanese, see Omura and Kizawa 2017; for Turkic, Levitskaya 1997a–c; for the borrowing of ‘silk’ from Sinitic into Mongolic into Turkic and Tungusic, see Shimunek 2017), historical comparative approaches of textile vocabulary across the Transeurasian languages are virtually non-existent. Archaeological studies on textile production in the Neolithic and Bronze Age in the Transeurasian-speaking region are somewhat more numerous and focus on specific regions such the Russian Far East (Hyland *et al.* 2002; Furusawa 2007; Kuzmin *et al.* 2012), Korea (Choi H 1985; Choi D-J 2011) and Japan (Fujimura 1985; Nunome 1985, 1995; Takeuchi 1985; Cort 1989; Ozeki 2018; Noshiro and Sasaki 2014). However, none of these studies provides a cross-regional comparison of textile technology.

Here we intend to fill a gap in the current literature, not only by comparing words and tools for textile production across North and East Asia, but also by integrating the linguistic and archaeological evidence in a single approach, for which we use the term ‘archaeolinguistics’. Other studies that have examined the connections between textile traditions, linguistic phylogenies and population histories in Eurasia include Buckley's (2012) research of the spread of Southeast Asian Neolithic weaving technologies in association with the expansion of the Austronesian languages and Tehrani *et al.*’s (2010) cultural evolutionary studies of Iranian tribal carpets. However, an integrative study examining parallels between early dispersal patterns of textile technology, farming, language and people has not yet been undertaken for North and East Asia. Through our holistic approach, we hope to remedy this situation and contribute to the current understanding of ancient population movements in North and East Asia.

## Methods

### Linguistic paleontology

First, it is necessary to look in somewhat more detail at the linguistic methods to be used in addressing the problem. We are dealing with the ancestral language of the Transeurasian languages, conventionally called ‘proto-Transeurasian’. This language is not attested in written records and is separated by several millennia from the earliest attested written sources of Old Turkic, Middle Mongolian, Jurchen, Middle Korean and Old Japanese. Nevertheless, it is possible to reconstruct this language by ‘unwinding’ regular and systematic similarities among the attested Transeurasian languages. In particular, we are concerned with the vocabulary of proto-Transeurasian as a way of determining the material culture of the speakers of that language, especially the ways in which it reflects the degree of familiarity with textile production. How can we tell what the vocabulary of proto-Transeurasian was like in this domain? Linguistic paleontology is a subfield of comparative historical linguistics that enables us to study human prehistory by correlating our linguistic reconstructions with information from archaeology about the possible cultural and natural environment of the speakers of the proto-language. The method was first introduced under this label by Adolphe Pictet (1859), but it is also known as ‘cultural reconstruction’ (Epps 2015) or ‘Wörter und Sachen technique’ (Campbell 2004 [1998], pp. 367–368). It relies on two assumptions, specifically, first, that words and their meanings can be confidentially reconstructed to the proto-language, and second, that reconstructed words allow us to make direct inferences about the culture of the ancient speech communities that used these words. However, the method is by no means foolproof. First, semantic reconstruction is generally less precise than phonological reconstruction. Second, the repurposing of words with a non-cultural meaning as words with a cultural meaning after the importation of a specific technology might erroneously lead us to attribute this word to the proto-language. For instance, an unattentive linguist might erroneously attribute the word ‘mouse’ in the meaning of ‘computer device’ to the proto-Germanic language because this meaning is disseminated across most of the contemporary Germanic languages. Third, if we can only reconstruct a single cultural item, say one word for ‘weaving’ that is not backed up by other members of its semantic domain, *in casu* textile production, there is reason for suspicion. Finally, as the traditional aphorism goes, ‘absence of evidence is not evidence of absence’. The observation that there is no proto-Transeurasian reconstruction for ‘hand’, for instance, would hardly lead to the conclusion that the ancestral speakers of Transeurasian lacked hands. In the same way, the lack of a reconstruction for ‘spindle whorl’ may be explained by the fact that the ancestral speakers were not familiar with spindle whorls, but it could also be due to the innovation and replacement of the common word for ‘spindle whorl’ over time. Nevertheless, when combined with the stringent checks and balances of the historical comparative method, linguistic paleontology is reasonably robust as a way of determining proto-culture through proto-language. Therefore, we will apply this method in order to make predictions about the familiarity with textile production of the ancestral Transeurasian speakers.

### Archaeolinguistic mapping

**Figure 2.**
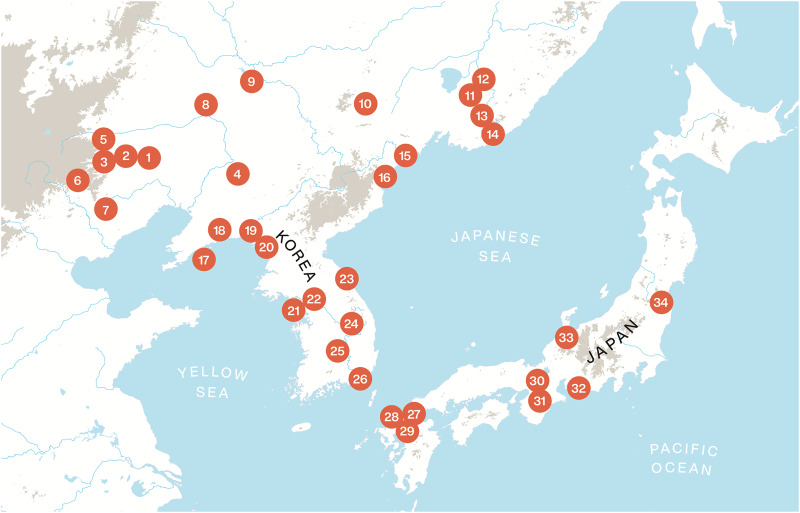
Location of the archaeological sites reviewed in this study: 1, Xinglongwa (6400–5200 BCE); 2, Zhaobaogou (5400–4500 BCE); 3, Baiyinchanghan II (5400–4500 BCE); 4, Xinle (5500–4800 BCE); 5, Jiefangyingzi (4500–3000 BCE); 6, Weijiawopu (4500–4000 BCE); 7, Niuheliang (4500–3000 BCE); 8, Haminmangha (4000–3000 BCE); 9, Zuojiashan (3000–2600 BCE); 10, Lower Yinggeling (2000 BCE); 11, Siny Gai A lower layer (ca. 2000–1300 BCE); 12, Bogolyubovka–1 (ca. 1900 BCE); 13, Sheklyaevo 7 Upper Zaisanovka horizon (ca. 2000–1300 BCE); 14, Valentin Peresheek (2600–1900 BCE); 15, Sŏp'ohang (ca. 3000 BCE); 16, Nongpodong (ca. 3000 BCE); 17, Xiaozhushan III (4500–3300 BCE); 18, Lower Houwa (4350–3000 BCE); 19, Sinam-li (ca. 3000 BCE); 20, Upper Kungsan-li (3100 BCE); 21, Jungsandong (2250–2140 BCE); 22, Amsadong (4230–1430 BCE); 23, Osan-li (5050–2230 BCE); 24, Pangokdong (2300–1500 BCE); 25, Pibong-li (5010–3050 BCE); 26, Tongsamdong (4740–1800 BCE); 27, Sasai (ca. 800 BCE); 28, Nabatake (ca. 900–400 BC); 29, Yoshinogari (ca. 500 BC–AD 300); 30, Karako-Kagi (ca. 500 BC–AD 300); 31, Shimonagata B (300BCE–300AD); 32, Ikegami (ca. 400 BC–AD100); 33, Shiraiwa (ca. 300 BCE); 34, Youkaijichikata (ca. 300 BCE).

Next, we will test these predictions through a comparative survey of textile technology in Northeast Asia in the Neolithic and Bronze Age. For this purpose, we will examine 34 archaeological sites that have evidence for spindle whorls covering the West Liao River area in Northeast China, the Russian Far East, the Korean Peninsula and the Japanese Islands from the Neolithic to the Bronze Age. The location and dating of these sites are presented in Figure 2.

Our aim is to examine whether the agricultural societies in this region had access to the textile technologies appearing in our linguistic reconstructions. We will therefore rely on a comparative analysis of existing data with regard to the presence of millet and hemp cultivation and the use of needles, awls, spindle whorls and loom weights. Our comparison of different sites at different time depths will lead to inferences about the route of dispersal of textile technology and to determining correlations and discrepancies with the dispersal route of agriculture. Mapping cultural dispersal on linguistic phylogeny, we will correlate these expansive processes inferable from the archaeological record with the linguistic spreads visible in Transeurasian language classification. In this way, archaeolinguistic mapping will serve to visualize to what extent the ancient dispersal routes of textile and agriculture overlap and mirror the spreads of the Transeurasian ancestral languages.

## Analysis

### Linguistic reconstructions

We may now turn to the evidence for the reconstruction of textile vocabulary to proto-Transeurasian. An overview of the linguistic cognates as well as a detailed dataset underlying our analysis can be found in Supplementary Table S1. Based on our dataset, we can reconstruct a verb for making rope, pTEA *nap- ‘to make rope’ and two verbs for sewing, pTEA *nup- ‘to sew’ and pTEA *sili-‘to sew’, but these may refer to activities connected to the production of textiles found at pre-agricultural sites and do not necessarily associate the speakers of proto-Transeurasian with a Neolithic society. Indeed, sewing and the manufacture of cordage do not need to imply weaving. The earliest actual textiles found preserved in East Asia so far are dated to the Early Neolithic Rudninskaya archaeological culture, 9400–8400 cal BP by AMS dating. Parts of ropes, nets and mats, produced without spindle-whorl technology, were found in the Chertovy Vorota Cave in the Primorye Province of the Russian Far East (Kuzmin *et al.* 2012). Impressions of cord and cloth were found in the earliest known pottery in the Russian Far East, for example at the Gromatukha site in the Middle Amur basin dated to around 12 500 BCE in the early Holocene (Hyland *et al.* 2002). Actual fragments of rope and string along with willow basketwork have been found at Angangxi, now a desert site in western Manchuria, ca. 6000–7000 BCE (Chang 1986, p. 64). Jomon pottery, some of the earliest pottery found in East Asia beginning around 14,500 BCE, is named for its cord-marked impressions (Kaner 2009; Matsuura 2017), although there is no evidence of any kind of plant cultivation at that time. In Japan, baskets are known from as early as 5760–5980 cal BCE at the Higashimyō site (Saga) (Kuzmin *et al.* 2012).

More strikingly, we can attribute verbs for weaving and spinning to proto-Transeurasian such as pTEA *pɔrɔ- ‘to weave (cloth)’ and pTEA *tɔmʊ- ‘to spin’, which seem to be related to more sophisticated processes of textile production in the Neolithic. Firm archaeological evidence of woven cloth and production tools for weaving and spinning begins later than that for rope and twine. Conical spindle whorls, ceramic disks described as spindle whorls and ‘net’ weights that in reality may be loom weights are often listed in site reports in Neolithic East Asia. Rope belts on otherwise nude figures and costumes depicted on figurines in the Hongshan period (4500–2900 BCE) provide another insight into Late Neolithic textile use (Childs-Johnson 2001, pp. 19–20).

The device associated with early textile production in Northeast Asia is the loom, either a horizontal back strap loom or a vertical warp weighted loom (Omura and Kizawa 2017; Kuzmin *et al.* 2012). The latter device, a wooden frame consisting of two vertical posts with a horizontal beam between them, required the attachment of net weights to the lower ends of plant fibers to stretch them out (Kent and Nelson 1976). The backstrap loom, which did not require weights, was more widespread in the Yellow River region and to the south of it.

We can further reconstruct a verb *giri- ‘to cut’ to proto-Transeurasian that seems to have been especially applied in the domain of textile production because the instrumental nouns derived from the relevant verbs in Old Japanese and Manchu both refer to an instrument restricted to cutting cloth and the corresponding Turkic verbs are specialized for the cutting of strips of pelt or cloth. In absence of scissors, stone knives were used to cut hemp and other products during the Neolithic in Northeast Asia (An 1955).

While the proto-Transeurasian textile vocabulary often gets lost in Turkic and Mongolic languages, it is well preserved in Japonic and Koreanic languages. In addition, there are three instances of common textile vocabulary restricted to proto-Japano-Koreanic, i.e. pJK *parʌ- ‘to sew’, pJK *pu- ‘to spin, twist (thread)’, pJK *pata- ‘to weave (cloth) with a loom’ and pJK *asa ‘hemp’. This seems to indicate that Transeurasian-speaking populations moving eastwards with agriculture retained more ‘traditional’ technologies such as textile production whereas those moving westwards were more exposed to west Eurasian technologies and replaced their vocabulary accordingly. Although archaeologists rarely find any differentiation between sites with agriculture and sites specializing in herding in terms of the appearance of spindle whorls, the site of Maoqinggou (740–345 BCE), just outside the Yan Wall in Manchuria, is an exception in that it shows significant differences in burials between agricultural and pastoral people. The tombs that exclusively contain spindle whorls are oriented north–south and imply an agricultural economy, while east–west-oriented graves lack spindle whorls and imply a pastoralist economy (Wu 2007). This may suggest that the herders were supplied with cloth by the agricultural weavers, while they may have been supplying wool to the weavers (Wu 2004, p. 229). Therefore, weaving seems to be associated more closely with agricultural people than with pastoral people living at the same time in the same region.

Genes are always inherited by offspring from their parents, and so are languages and material culture in many cases, but not always because social phenomena may disrupt the horizontal transmission. Tools for textile production are expected to become vertically transmitted from parents to their offspring along with the words referring to them. However, sometimes these traditions may be acquired from sources other than the immediate ancestors by way of horizontal transmission, for instance, through cultural influence from neighboring populations. Linguistically, we can exclude borrowing with a high degree of probability for the etymologies above. Owing to a number of linguistic indications such as the regularity of sound correspondence, the lack of morphological segmentability of the involved comparanda, the multiple rather than binary comparative setting including up to five daughter branches and the solid distribution of cognates in individual daughter branches, these etymologies are considered to be relatively borrowing-proof. However, a few comparative sets relating to textile technology are indicative of borrowing rather than inheritance because they display problematic sound correspondences, are morphologically segmentable, have a limited distribution in some daughter branches or match a probable donor word outside the family. This may be the case for both cognate sets for ‘hemp’ included in Supplementary Table S1.

The high tone of MK *·sam* ‘hemp’ indicates the loss of an initial vowel and justifies the reconstruction of *asam ‘hemp (*Cannabis sativa*)’ to proto-Japano-Koreanic. However, the attestation of an Old Indo-Aryan *śaṇ* ‘hemp’ in the ancient religious hymns of the Rigveda from the second millennium BCE, which goes back to an even older Scythian form *sana ‘hemp’ (Mayrhofer 1992–2001, p. 605; Gamkrelidze and Ivanov 1995, p. 570) may indicate that this common form is ultimately borrowed from the west. Contact may also explain the correlations yielding the reconstruction of proto-Altaic *olo ‘hemp’ because the form has a limited distribution in Tungusic: it is restricted to Manchu only and may be borrowed from Mongolic. As Manchu does not reflect the Mongolic collective suffix *-*sun* the borrowing should precede proto-Mongolic and have taken place not later than the first millennium AD. There are different hypotheses on the origin of hemp domestication, of which the two most frequently cited refer either to China (Crawford 2006) or Central Asia (Russo 2007) as the domestication center. From the third millennium onwards, there is an increase in evidence of *cannabis* remains from East Asia, which may be associated with an exchange network through the Eurasian steppe zone (Long *et al.* 2017). The borrowing of the words for hemp may be seen in this context. The current evidence suggests that the use of fibers from wild plants preceded the cultivation of plants such as hemp for this purpose. Sarah Nelson (2017) infers that at the Amsadong site in the Early Korean Neolithic wild plants were used for weaving and medicine as well as unspecified cultivated plants. Lee Gyoung-Ah (pc 01.09.2019) reidentified hemp discovered earlier at the Neolithic Taych'on-li site in Ch'ungch’ŏng province as a soil clump. The current stage of research is such that there are no hemp remains prior to the Early Iron Age in Korea. In Japan, however, small-scale cultivation of hemp probably dates back to the Early Jomon period (Kudo and Hitoki 2014; Noshiro and Sasaki 2014).

Ramie, a plant that is native to Northeast Asia was a widespread fiber in Neolithic and Bronze Age that was used for textile production and subject to intentional soaking. However, we do not find comparative sets that are indicative of a common ancestral word for this plant.

Surprisingly, at least three different verbs for ‘soaking’ can be reconstructed for Transeurasian, pTEA *simi- ‘to soak’, pTEA *ulu- ‘to soak, wet’ and pTEA *nɔr- ‘to soak’ in addition to *deb- ‘to soak’ in proto-Altaic and *kam- ‘to wash, soak’ in proto-Japano-Koreanic. Why did the ancestral speakers of Transeurasian need so many different verbs for soaking? One explanation could be connected to food production, for instance, in the Neolithic, nuts were soaked to remove the tannic acid (Kawashima 2016). However, as a number of soaking verbs developed secondary meanings such as ‘to stain’, ‘to paint’ and ‘to bleach’ or derived nouns meaning ‘clothes’, some may be connected to textile production. As evident from the present-day commercial production process in Northeast Asia, tree bark, ramie and hemp need to be soaked before they can be turned into thread, and hemp strips and thread are repetitively soaked and heated in the process of weaving (Clarke 2006).

### From linguistic inferences to archaeological predictions

In addition to the availability of agricultural vocabulary, Robbeets (2017, 2020a) used the linguistic dating and the location of the ancestral nodes in the Transeurasian family to associate ancestral languages with archaeological cultures. Bayesian phylogenetic analysis infers the time depths of separation at 4700 BCE for proto-Transeurasian, 3293 BCE for proto-Altaic, 1552 BCE for proto-Turko-Mongolic and 1850 BCE for proto-Japano-Koreanic (Robbeets and Bouckaert 2018). Different methods for the determination of linguistic homelands converge on situating proto-Transeurasian, proto-Altaic and proto-Turko-Mongolic in the West Liao River region, proto-Tungusic in the southern part of the Primorye region of the Russian Far East and proto-Japano-Koreanic on the Liaodong Peninsula before the individual Transeurasian languages reached their present-day locations. The connections in space, time and subsistence mode suggest an association between language families and archaeological cultures, as presented in Supplementary Table S2.

This association between ancestral languages and archaeological cultures leads to at least three predictions. First, since the speakers of proto-Transeurasian used vocabulary for weaving and spinning, we infer that they were familiar with these techniques. This gives rise to the expectation to find evidence for weaving and spinning in the Xinglongwa (6200–5400 BC) and Zhaobaogou (5400–4500 BC) cultures in the West Liao River region, such as loom weights and spindle whorls.

Second, since Transeurasian textile vocabulary solidly survived in the Japano-Koreanic and Tungusic subgroups, we infer that the speakers of Transeurasian took their knowledge of spinning and weaving with them on their journey to the southern part of the Primorye and the Liaodong Peninsula and from there over the Korean Peninsula to Japan. We therefore predict a connection between Neolithic spindle whorls in the Liao River Basin and those of the Neolithic on the Liaodong Peninsula, the southern part of the Primorye region and the Korean Peninsula as well as Bronze Age Japan.

Third, since the ancestral Transeurasian proto-languages display cognate textile vocabulary in addition to agricultural vocabulary, we infer that words for textile and agriculture were spread simultaneously by the early speakers. We predict that Neolithic and Bronze Age cultures with evidence for spindle whorls will tend to preserve evidence for agriculture and that we cannot find spindle whorls preceding agriculture in North and East Asia.

## Results

### Prediction 1: Earliest evidence for spindle whorls in the Transeurasian linguistic region goes back to the Xinglongwa and Zhaobaogou cultures

In order to test these predictions, we examined the Neolithic and Bronze Age archaeological sites in Northeast Asia with evidence for textile technology presented in Figure 2. Table 1 gives an overview of these sites and specifies whether there is evidence for millet cultivation, agricultural tools, hemp cultivation, spindle whorls and bone needles or awls. The feature ‘agricultural tools’ refers to the discovery of stone, clay or wooden artifacts that are assumed to have been used in agricultural activities, such as plowing and harvesting.

**Table 1.** Selection of sites with textile technology in the Transeurasian linguistic region, specifying the simultaneous occurrence of millet or hemp cultivation and agricultural tools: 0 means ‘absent’, 1 means ‘present’ and ‘?’ marks uncertainty. The indicated number of the sites corresponds to their location on the map in Figure 2

Region	Culture	Site no.; Figure 2	Site name/date	Millet cultivation	Agricultural tools	Hemp cultivation	(Bi)conical spindle whorl	Flat spindle whorl	Bone needle/awl
	*Northeast China*								
Liao River	Xinglongwa 6400–5200 BCE	1	Xinglongwa 6400–5200 BCE	1	1	0	0	1	1
Liao River	Xinglongwa 5400–4500 BCE	3	Baiyinchanghan II 6400–5400 BCE	1?	1	0	1	1	1
Liao River	Zhaobaogou 5400–4500 BCE	2	Zhaobaogou 5400–4500 BCE	0	1	0	0	1	1
Lower Liao River (Shenyang)	Xinle 5200–4000 BCE	4	Upper Xinle 4800–4000 BCE	1?	1	0	1	1	1
Liao River	Hongshan 4500–3000 BCE	5	Jiefangyingzi 4500–3000 BCE	No data	No data	No data	1	1	No data
Liao River	Hongshan 4500–3000 BCE	6	Weijiawopu 4500–4000 BCE	1	1	0	0	1?	0
Liao River	Hongshan 4500–3000 BCE	7	Niuheliang 4500–3000 BCE	0	1	0	0	1?	1
Liao River	Haminmangha 3500–3000 BCE	8	Haminmangha 4000–3000 BCE	1	1	1	0	1	1
Amur River	Zuojiashan 4000–600 BCE	9	Zuojiashan 2600–3000 BCE	1	1	0	0	1	1
Amur River	Yinggeling 4000–1000 BCE	10	Lower Yinggeling 2000 BCE	0	1	0	1	1	1
Yalu River	Xiaozhushan (I–V) 4700–2100 BCE	17	Xiaozhushan III 4500–3300 BCE	1	1	0	0	1?	1
Yalu River	Houwa 4500–3000 BCE	18	Lower Houwa 4350–3000 BCE	0	1	0	0	1	1
	*Southern part of Primorye Province of Russian Far East*								
Khanka–Ussuri Plain, east of Lake Khanka	Zaisanovka 3200–1500 BCE	11	Siny Gai A– lower layer ca. 2000–1300 BCE	No data	1	No data	1	1	1
Eastern seacoast	Zaisanovka 3200–1500 BCE	14	Valentin–Peresheek 2900–2300 BCE	No data	1	No data	1	1	0
East of Khanka–Ussuri Plain	Zaisanovka 3200–1500 BCE	13	Sheklyaevo 7 Upper Zaisanovka horizon ca. 2000–1300 BCE	1	No data	0	0	1	No data
Khanka–Ussuri Plain, south of Lake Khanka	Zaisanovka 3200–1500 BCE	12	Bogolyubovka–1 Ca. 1900 BCE	1	1	0	0	1	No data
	*Korean Peninsula*								
Yangyang, Kangwŏndo	Early Chulmun 6000–3500 BCE	23	Osan-li 5050–2230 BCE	0	1	0	1	0	No data
Changnyŏng, South Kyŏngsang	Early Chulmun 6000–3500 BCE	25	Pibong-li 5010–3050 BCE	1	1	0	1	1	No data
Seoul	Early–Middle Chulmun 6000–2000 BCE	22	Amsadong 4230–1430 BCE	1	1	0	1	1	No data
Tumen River	Middle–Late Chulmun 3500–1500 BCE	15	Sŏp'ohang	0	1	0	1	0	0
Tumen River	Middle–Late Chulmun 3500–1500 BCE	16	Nongpodong	0	1	0	1	0	No data
Yalu River	Middle–Late Chulmun 3500–1500 BCE	19	Sinam-li 3000 BCE	0	1	0	1	1	0
Taedong River	Middle–Late Chulmun 3500–1500 BCE	20	Upper Kungsan-li 3100 BCE	1?	1	0	1	1	1
Nakdong River, Pusan	Middle–Late Chulmun 3500–1500 BCE	26	Tongsamdong 4740–1800BCE	1	1	0	1	1	1
Incheon	Late Chulmun 2000–1500 BCE	21	Jungsandong 2250–2140 BCE	1	1	0	1	1	No data
Wonju, Kangwŏndo	Late Chulmun 2000–1500 BCE	24	Pangokdong 2300–1500 BCE	1	1	0	1	0	No data
	*Japanese Islands*								
Fukuoka	Yayoi 900BCE–300AD	27	Sasai ca. 900 BC–600BCE	0	1	0	0	1	1
Saga	Yayoi 900BCE–300AD	28	Nabatake ca. 900–400 BCE	1	1	0	0	1	1
Saga	Yayoi 900BCE–300AD	29	Yoshinogari ca. 500 BCE–AD 300	0	1	1	0	1	0
Nara	Yayoi 900BCE–300AD	30	Karako-Kagi ca. 500 BCE–AD 300	0	1	0	0	1	0
Osaka	Yayoi 900BCE–300AD	31	Ikegami ca. 400 BCE–AD100	0	1	0	0	1	0
Shizuoka	Yayoi 900BCE–300AD	32	Shiraiwa ca. 200 BCE–AD 200	0	1	0	0	1	0
Ishikawa	Yayoi 900BCE–300AD	33	Yōkaichijikata ca. 300 BCE–AD 200	0	1	0	0	1	1
Fukushima	Yayoi 900BCE–300AD	34	Shimonagata B ca. 300 BCE–AD 300	0	0	0	0	1	0

Spindle whorls indeed appear as early as the Xinglongwa culture, the first farming culture in Northeast China. They continue through the Zhaobaogou culture into the Hongshan culture, also covering outlying Neolithic cultures such as Haminmangha on the eastern plains of the West Liao River, Xinle in the Lower Liao River basin and Houwa and Xiaozhushan on the Liaodong Pensinula. Sites in Jilin dating to the third millennium BCE also display spindle whorls.

The evidence for spindle whorls continues into the Russian Far East in the third and second millennia BC, where they appear at the Neolithic sites of Valentin-Peresheek (Andreeva, 1987; Zhushchikhovskaya, 2006), Siny Gai A lower layer (Zhushchikhovskaya, 2006; Brodyansky, 2013), Sheklyaevo-7 Upper Zaisanovka horizon (Klyev *et al.* 2003) and Bogolyubovka-1 (Sergusheva, 2013; Garkovik and Sergusheva, 2014). These sites represent different local variants of the Zaisanovka cultural tradition. Most datings are marked as ‘ca.’, indicating that there is no carbon dating available for the sites. The Valentin-Peresheek and Siny Gai A preserve evidence for conical, biconical and flat disk-like spindle whorls. The Bogolyubovka-1 site, which is located not far from Siny Gai, produced a series of earthenware flat disks similar to the Siny Gai A ones. Sheklyaevo-7, east from Siny Gai, is a small-sized settlement with complicated stratigraphy, but the flat disk-like whorls in the Upper Zaisanovka horizon are interpreted as late Neolithic, Zaisanovka culture. Although the Siny Gai and Valentin-Peresheek sites belong to different landscape-climatic zones, the temporal difference between them is insignificant. In general, the artifact assemblage from Siny Gai looks somewhat more developed than the one from Valentin-Peresheek.

For the Korean Neolithic, spindle whorls are reported in northern as well as southern sites. Most sites are dated to the Middle to Late Chulmun period but the water-logged site of Pibong-li in southeastern Korea (Kimhae National Museum 2018), the Amsadong site (Nelson 2017) and the Osan-li site are dated to the Early Chulmun period. Pibong-li adds details about early use of plants for textiles. Bags woven from grass were found near a piece of a wooden boat. The dates are 4670 ± 60 BCE, making the site as old as Amsadong and showing that plant usage for weaving occurred even in the south of the Korean peninsula in the Early Neolithic. Further studies of seeds will expand our knowledge of cultivation at this important site, but in terms of textile production, many spindle whorls, needles and weights demonstrate a lively industry. This site, being close to Japan, may be a place from which textiles spread. Finds of weights and spindle whorls in Early (1300–800 BCE) and Middle Mumun (800–500 BCE) show that virtually every site contains spindle whorls as well as weights in Bronze Age Korea (Kim 1978; Lee 2019).

The first evidence of woven cloth in Japan is thought to have appeared in the early part of the Yayoi period (900 BC–AD 300) when spinning and weaving technologies were brought from Korea along with an agricultural package including rice and millets. Small-scale cultivation of hemp probably dates back to the Early Jomon period but ramie and silk are also known from Yayoi contexts. Nagasaki (1978) proposed that the perforated circular disks known from the Jomon period served as spindle whorls and this remains a possibility. Yayoi-period spindle whorls were made from clay, stone, wood or bone and antler, but those of clay or stone were the most common. Most Yayoi spindle whorls are flat and undecorated disks. However, a few incised decorations are known from Nabatake (Saga), Magarita (Fukuoka) and Shimonogata B (Fukushima). Conical spindle whorls were absent in the Yayoi but common in the Kofun period (AD 250–700). Wooden spindles are known from several Yayoi sites. At Kitoragawa (Osaka) a wooden spindle was found in association with a stone spindle whorl. Wooden parts of looms have been found at a number of Yayoi sites including Karako-Kagi and Toro (Takeuchi 1985). Needles from Yayoi contexts include one made from a sea urchin spine from Nabatake, and those made from bone from Asahi (Aichi), Ayaragi (Yamaguchi), and Kitoragawa.

In sum, although the attestation of spindle whorls in the southern part of the Korean Peninsula around 4600 BCE is strikingly early, the earliest evidence for spinning technology in the Transeurasian-speaking region precedes this date by about a millennium and indeed comes from the Xinglongwa and Zhaobaogou cultures in the West Liao River basin.

### Prediction 2: Connection between Neolithic technology for spinning and weaving in the Liao River Basin and those in the southern part of the Primorye and on the Korean Peninsula as well as those in Bronze Age Japan

Among the evidence for Neolithic and Bronze Age technology for spinning and weaving in Northeast Asia, we find conical or biconical spindle whorls, disk-like whorls and loom weights. A spindle whorl is a spherical tool that is fitted onto a spindle to increase and maintain the speed of the spin when twisting fibers and making threads. The spindle itself is a rounded, usually wooden, rod for twisting into thread the fibers pulled from the basic material. Suspended from the thread that is being spun, the spindle and whorl are controlled by the spinner. While there is no doubt that (bi)conical spindle whorls were used for spinning, the exact function of disk-like whorls, i.e. holed, flat disk-like objects, usually made from ceramic or stone, is not entirely clear. They may have been fitted individually onto the spindle as a whorl, but they may also have been used in conjunction, in hanging position as a system. Loom weights are ceramic or stone weights tied at the bottom of warp threads in a vertical loom to create an even tension and give greater control to the weaver.

**Figure 3.**
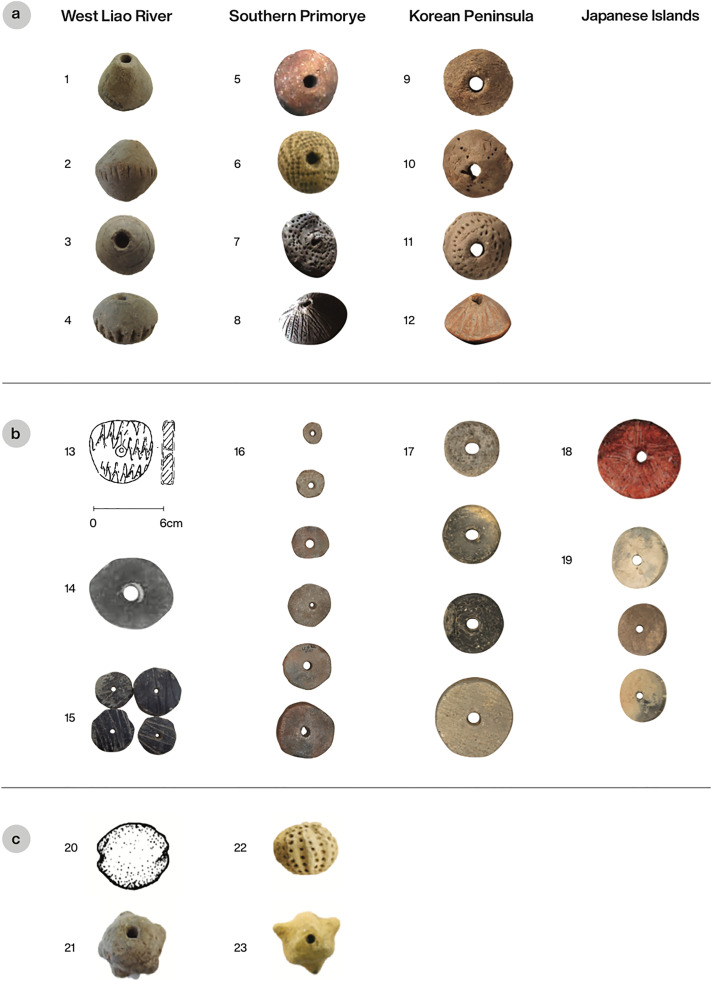
Comparison of spinning and weaving technology in Neolithic and Bronze Age Northeast Asia. (a) Conical ceramic spindle whorls: 1–4, Hongshan, Wengniute Banner, Inner Mongolia (http://blog.sina.cn); 5–6, Zaisanovskaya, Lower Siniy Gai A, southern Primorye (Museum of Archaeology and Ethnography of Far Eastern Federal University); 7–8, Zaisanovskaya, Valentin Peresheek, southern part of Primorye (Institute of History, Archaeology and Ethnography of Peoples of Far East); 9, Early Chulmun, Osan-li, Korea (Chuncheon National Museum); 10, Middle Chulmun, Hasidong, Korea (Chuncheon National Museum); 11, Late Chulmun, Bangokdong, Korea (Chuncheon National Museum); 12, Late Chulmun, Wonsudae, Korea (National Museum of Korea). (b) Disk-like whorls: 13, Xinglongwa (Schelach and Teng 2013, p. 44); 14, Zhaobaogou; Aohan Banner (Aohan Zhaobaogou, Xinshiqi Shidai Juluo 1997, Fig. 35); 15, Hongshan, Wengniute Banner, Inner Mongolia (http://blog.sina.cn); 16, Zaisanovka, Lower Siny Gai A, southern part of Primorye (Museum of Archaeology and Ethnography of Far Eastern Federal University); 17, Early–Middle Chulmun, Amsadong, Seoul (National Museum of Korea); 18, Yayoi, Shimonagata B, Japan; 19, Yayoi, Karako-Kagi (http://www.town.tawaramoto.nara.jp/karako_kagi/museum/syoukai/search/2/yayoinowaza/oruamuwaza/oruwaza/bosuisha/7569.html). (c) Loom weights: 20, Xinglongwa (Wu and Liu 2003); 21, Hongshan, Wengniute Banner, Inner Mongolia (http://blog.sina.cn); 22–23, Zaisanovskaya, Lower Siniy Gai A, southern part of Primorye (Museum of Archaeology and Ethnography of Far Eastern Federal University).

Figure 3 compares the spinning and weaving technology from the Neolithic and Bronze Age in the West Liao River region, the southern part of the Primorye region, the Korean Peninsula and Japan. The spindle whorls in Figure 3a are similar in that they have a conical shape with a hole in the middle, are made from clay, are decorated with similar radiating or concentric patterns using pitting or incised line techniques and measure about 6 cm in diameter. Most examples in Figure 3a have a three-dimensional conical shape, tapering smoothly from a flat circular base to a point, but biconical shapes, looking like two cones with their bases together, such as the Hongshan whorl in 2–3, are also found in the southern part of the Primorye and the Korean peninsula (Furusawa 2007, pp. 86–88). Whereas some spindle whorls, such as those in 1, 5 and 9, do not reflect any ornaments, others such as those in 3, 7 and 11 display a concentric, spiral-like design, while yet others, such as those in 4, 8, 10 and 12 form a pattern that radiates away from the center. The decorations in 6, 7, 10 and 11 are made by creating small holes, using a pitting technique, while those in 2, 3, 4, 8 and 12 display an incised line technique.

The whorls in Figure 3b are similar in that they have a flat, disk-like shape with a hole in the middle, remain largely undecorated, are often but not always made from earthenware, come in sets of different sizes and are often reused from pottery fragments. Except for the Z-patterns on the whorls 1–3 from the Liao River Basin and the radiating incised lines on whorl 18 from Shimonagata B in Japan, most of the flat whorls remain undecorated. In addition to clay, they can also be made from stone or bone, such as the stone Hongshan whorls in 15 and some whorls belonging to the Zaisanovskaya culture (Furusawa 2007, p. 87). The whorls in 16–18 range between 2 and 6 cm in size and seem to form sets, which raises the possibility of them being used jointly in hanging position, perhaps as a kind of pulley system.

The ceramic weights in Figure 3c most probably are textile-producing equipment. From the Xinglongwa period (6200–5400 BC) onwards, stone weights are common in the Neolithic but usually these are reported without discussion as ‘net sinkers or loom weights’ (Wu and Liu 2003). However, these objects were arguably loom weights, as they have been more often found in houses rather than on shores where nets are kept, and they may be associated with spindle whorls. For example, small flat pebbles with notches in both ends have been found at Amsadong in Korea, where an argument could be made that that they are evidence of weaving rather than net-fishing, because the fish remains from that site are unimpressive (Nelson 1975; Kent and Nelson 1976). The loom weights can either have a hole in the middle, such as the weights in 21 and 23, or lack one, such as the weights in 20 and 22. They can be made from stone, such as the Xinglongwa weight in 20, but they are more generally made from earthenware, such as the weights in 21–23, because clay gives the weaver greater control over its shape, size and weight and is easier to pierce than rocks or pebbles.

In sum, there appear to be significant similarities between (bi)conical spindle whorls, disk-like whorls and loom weights across different regions of Northeast Asia at different times in the Neolithic and Bronze Age.

**Figure 4.**
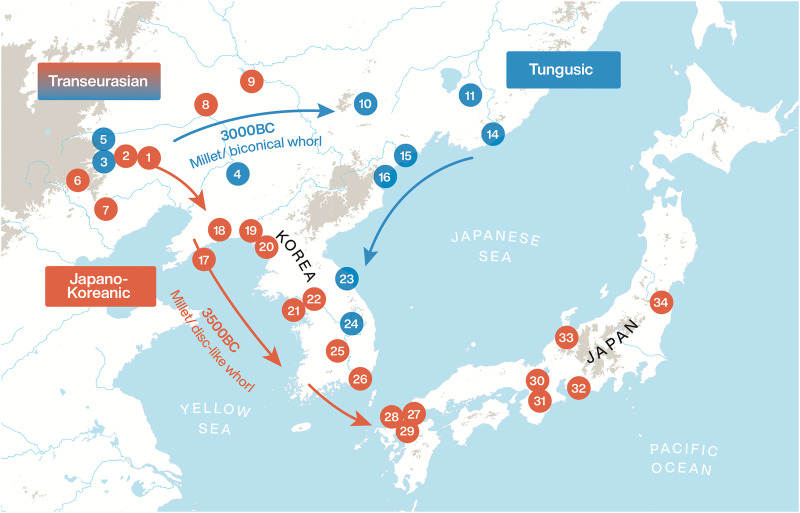
The dispersal of textile technology, agriculture and language across Northeast Asia. The numbers correspond to the sites, listed in Figure 2. Archaeological sites where (bi)conical whorls have been recovered in relatively high proportions are indicated in blue.

In addition, there is an interesting contrast in the distribution of (bi)conical whorls as opposed to disk-like whorls. There is a strong concentration of disk-like whorls in the Xinglongwa and Zhaobagou cultures of the West Liao River Basin and they are exclusively found in the Xiaozhushan and Houwa cultures of the Liaodong Peninsula (Furusawa 2007, p. 100) and in Yayoi Japan. They are also more frequent than (bi)conical whorls in Neolithic sites of the western and southeastern part of the Korean Peninsula (Choi 2011). In contrast, the Xinglongwa and Hongshan cultures of the West Liao River Basin yield biconical whorls at a few sites, such as the Baiyinchanghan II and Jiefangyingzi sites in Wengniute Banner. For the sites of Niuheliang and Weijiapou a question mark is inserted with disk-like whorls because the site reports mention spindle whorls without picture or specification of their being disk-like or (bi)conical. However, as (bi)conical whorls seem to be the exception rather than the norm here, we would expect excavators to make mention of (bi)conical shapes. Interestingly, the Upper Xinle site in the Lower Liao River Basin, the Yinggelin site on the Amur River and the Zaisanovka culture of the southern part of the Primorye region display (bi)conical spindle whorls. They are also more numerous than disk-like whorls in the northeastern and mid-eastern part of the Korean Peninsula (Choi 2011, Nelson 1993, pp. 100, 160). This observation hints at two geographically and chronologically different routes for the dispersal of textile technology out of the Liao River region, indicated in red and blue in Figure 4.

### Prediction 3: Neolithic/ Bronze Age cultures with evidence for spindle whorls tend to have evidence for agriculture

This prediction can be confirmed. The majority of sites with evidence for spindle whorls in Table 1 score positive for the attestation of millet cultivation or agricultural tools. Four sites in Northeast China lack direct evidence of millet cultivation but they all have indirect evidence of agriculture such as stone hoes and axes.

Unfortunately, the Valentin-Peresheek and Siny Gai sites from the Russian Far East were excavated in the 1960s and 1970s, when soil flotation for detecting of plant remains was not yet applied. Therefore, we do not have any archaeobotanical evidence for millet or hemp cultivation from these sites, but the Zaisanovka culture to which these sites belong is known as the first farming culture in the Russian Far East. Carbonized millet grains have been found at the Bogolyubovka-1 site and at the upper Zaisanovka horizon of the Sheklyaevo-7 site (Leipe *et al.* 2019). In addition, the Siny Gai A and Bogolyubovka-1 sites yield stone constricted hoes, grinding slabs and grinders, suggesting agriculture. At the Valentin-Peresheek site a series of rough hoes and small-sized grinding slabs with grinders were excavated, but analysis shows that these were used for crushing of the mineral pigment ocher. It is unclear whether they were used for agricultural purposes as well. However, the Valentin-Peresheek area, which is located at the seacoast, offers less favorable conditions for agriculture than the Siny Gai area, located close to Lake Khanka in the southwestern part of the Primorye.

While the early site of Amsadong has provided most of the inferences about food production in the Early Neolithic of Korea, charred foxtail millet seeds have been systematically identified at Pibong-li as well (Lee and Kwak 2019). Osan-li depended heavily on fishing, and acorn remains along with pestles and mortars show that this was supplemented by gathering wild plants, but there is no evidence of millet cultivation (Ahn *et al.* 2015). Some Middle to Late Chulmun sites such as Sŏp'ohang, Nongpodong and Sinam-li lack direct evidence for millet cultivation, but agricultural tools have been discovered at these sites. Whereas the Kungsan-li site yields small amounts of unidentified grains, the Tongsamdong site has direct evidence for foxtail and broomcorn millet agriculture (Lee 2011). Flotation of Late Chulmun sites such as Jungsandong has yielded foxtail and broomcorn millet seeds (Kim *et al.* 2015). In general, all Korean Neolithic sites in the table yield various tools related to food production but it is hard to define them as exclusively agricultural tools. Half-moon-shape sickles, for instance, were probably used for harvesting, but could have been used for collecting wild edible plants too.

Cultivated plant remains were identified at only a few of the Japanese sites examined here, but this can be explained by the lack of systematic archaeobotanical investigations at those sites. There is no question that many of the sites from Japan with evidence of weaving and textile production are well known as important Yayoi period settlements with evidence for agriculture and for continental cultural traits such as rice paddy fields, defensive moats and palisades, wooden and stone farming tools, early bronze and iron, and carp aquaculture (Hudson 1990; Nakajima *et al.* 2019). At Yoshinogari (Saga), for example, at least six spindle whorls and 11 pieces of cloth made from both hemp and silk have been found (Shichida 2005; Hudson and Barnes 1991). Yoshinogari has also produced extensive evidence of an agricultural economy including reaping knives, forks and spades, mortars and pestles, and a series of large buildings which are reasonably interpreted as storehouses. Phytoliths of rice and barley have been identified at Yoshinogari but the archaeobotany of the site remains poorly understood. However, this does not mean that the inhabitants of the site did not practice farming.

## Discussion

Whereas pre-agricultural societies such as the Boisman (4825–2470 BCE) in the Russian Far East, the Incipient Chulmun (8000–6000 BCE) populations on the Korean Peninsula and the Jomon (10000–900 BCE) in Japan showed no evidence for weaving, spindle whorls appear with the transition to agriculture. We find the earliest evidence for spinning in Northeast China within the context of the Xinglongwa and Zhaobagou cultures in the West Liao River Basin. Subsequently, we find evidence for textile technology more sophisticated than sewing tools, starting from Zaisanovka times in the Russian Far East, Early Chulmun times on the Korean peninsula and Yayoi times in Japan. All of these cultures are associated with the beginning of agriculture in the respective regions.

The transition to sophisticated textile technology seems to mirror the transition to agriculture in time and space: around 3000 BCE millet agriculture was transmitted from the Hongshan culture in the West Liao River Region to the southern part of the Primorye (Li *et al.* 2020), while conical spindle whorls, disk-like whorls and loom weights similar to those of the Hongshan culture arrived in the southern part of the Primorye around the same time in the context of the same Zaisanovka culture. This route is indicated in blue on the map in Figure 4. Around 4000 BCE millet agriculture was transmitted from the Hongshan culture to the cultures on the Liaodong Peninsula (Xu 1995) and subsequently, around 3500 BCE, to the Korean Peninsula (Lee 2011). From there, an integrated agricultural package including millet and rice was further transferred to Japan in 900 BCE. Disk-like whorls and loom weights recalling those of the Xinglongwa culture appear around the same time as millet agriculture in the same sites on Liaodong, the Korean Peninsula and Japan. This route is indicated in red on the map in Figure 4.

Interestingly, the red and blue dispersal routes on the map also have a linguistic correlate: the ancestral proto-Transeurasian language has its homeland in the West Liao River region, the Japano-Koreanic branch separated from the Altaic unity around 4700 BCE and settled on the Liaodong Peninsula and the separation and dispersal of Koreanic and Japonic to their present-day locations followed in the ensuing millennia. The time and space of these linguistic dispersals mirror those of millet agriculture with disk-like whorls. in contrast, the Tungusic branch separated around 3300 BCE and moved from the West Liao River region to the southern part of the Primorye, mirroring the dispersal of millet agriculture with conical whorls.

As far as Transeurasian prehistory is concerned, we traced population movements in North and East Asia through the linguistics and archaeology of textile production. We found that the area West of the Liao River in Northeast China was a Neolithic center of diffusion, not only of millet agriculture, but also of words and tools for spinning and weaving. As such, we provided additional support for the Language/Farming Dispersal Hypothesis of the Transeurasian languages.

From a theoretical viewpoint, our study may have some broader implications for debates surrounding the Language/Farming Dispersal Hypothesis. By focusing on industries like textile production rather than just food production, we viewed the spread of agriculture as an interconnected assemblage of ideas, practices and technologies that travel together rather than as a mere collection of crops and tools associated with plant cultivation. From this perspective, the spread of textile technology becomes useful as a marker of agricultural dispersal and population migration. In line with Tehrani *et al.*’s (2010) assumption that the history of weaving traditions is strongly correlated with population histories and with Buckley's (2012) findings about the associated spread of Austronesian language with Neolithic weaving and farming technologies, our results show that parallels exist between the dispersal patterns of Neolithic technologies such as textile production and agriculture and the spread of the Transeurasian languages across Northeast Asia.

## Data Availability

All data and material underlying our research are referenced in the article or made available in the Supplementary Material.

## Supplementary material

For supplementary material accompanying this paper visit http://dx.doi.org/10.1017/ehs.2020.4. click here to view supplementary material
