# Supplementary material for: Tracing population movements in ancient East Asia through the linguistics and archaeology of textile production
Source: Evol Hum Sci. 2020 Feb 14;2:e5. doi: 10.1017/ehs.2020.4 (PMC10427276; doi:10.1017/ehs.2020.4)
Supplement: Supplementary file 1 [file S2513843X20000043sup001.docx]

**Tracing population movements in ancient East Asia**

**through the linguistics and archaeology of textile production**

Sarah Nelson, Irina Zhushchikhovskaya, Tao Li, Mark Hudson & Martine Robbeets

Supplementary Table S1

**Table S1.** Reconstruction of textile vocabulary to proto-Transeurasian and daughter branches based on linguistic comparison of the Transeurasian languages; MRCA most recent common ancestor, pTEA proto-Transeurasian, pA proto-Altaic, pJK proto-Japano-Koreanic, pTk proto-Turkic, OT Old Turkic, pMo proto-Mongolic, MMo. Middle Mongolian, WMo. Written Mongolian, pTg proto-Tungusic, Ma. Manchu, pK proto-Korean, MK Middle Korean, pK proto-Japonic, OJ Old Japanese

| MRCA | Japonic | Koreanic | Tungusic | Mongolic | Turkic |
| --- | --- | --- | --- | --- | --- |
| pTEA **nap*-  ‘to make rope’ | OJ *nap*-  ‘twist, plait (into rope)’  < pJ **nap*- | K *nah*-  ‘spin, make yarn’  < pK **nap*- | Oroch *lappi*  ‘tiers, straps’  <pTg **nap-ki* |  |  |
| pTEA **nup*-  ‘to sew' | OJ *nup*-  ‘to sew, stitch’  < pJ **nup-* | MK *nwu(·)pi*- ‘to sew, quilt’  < pK **nupi*- | Evenki *lupa*- ‘to prick’  < pTg **nup*- |  |  |
| pTEA **sili*-  'to sew’ |  | MK ̈ *sil* ‘thread’  < pK **sil(i)-i* | Nanai *sera*-  ‘to sew together’  < pTg **sira*- | WMo. *siri*-  ‘to quilt, stitch’  < pMo **siri*- |  |
| pTEA **pɔrɔ*- ‘to weave (cloth)’ | OJ *oro_2_s*- ‘deign to weave’  < pJ **orə*- | MK ¨*wol* ‘strand of rope, warp’  K *olk*- ‘to tie up, weave’  < pK **olʌ* | Ma. *foro*-  ‘to spin, turn’  < pTg **poro*- | MMo. *hura*-  ‘to tie around, wrap’  < **poro-* | OT *ör*-  ‘to plait (hair or fibers), weave’  Khalaj *hör*-  ‘to plait’  < **pö:r*- |
| pTEA **tɔmʊ*- 'to spin’ | OJ *tumu* 'spindle'  < pJ **tumu* |  | Even *tum*-  ‘to spin, wind’  < pTg **tomu*- | MMo. *tomu-*  *‘*to spin thread or rope’  < pMo **tomu-* |  |
| pTEA **giri*- 'to cut (cloth)' | OJ *ki_1_r-*  ‘to cut’  OJ *ki_1_ri* 'awl, pointed tool for piercing cloth’  < pJ **kira-* |  | Ma. *giri*-  ‘to cut a strip’  *giriku*:  ‘small knife for trimming skin, and cloth’  < **giri*- |  | OT  ‘to strip (hair), cut off, scrape’  < pTk **kïr*- |
| pTEA **simi*- ‘to soak’ | OJ *sim*-  ‘soak, permeate’  OJ *simi* ‘stain’  < pJ **sima*- |  |  | WMo. *sime*-  ‘to soak in, be saturated’  < pMo **sime*- |  |
| pTEA **ulu*- ‘to soak, wet’ | OJ *urum-*  ‘to get wet, moist’  < pJ **uru*- | MK *wuli*-  ‘to soak, bleach’  < pK **uli*- | Evenki *ula*- ‘to soak, wet’  < pTg **ula*- |  |  |
| pTEA **nɔr*- ‘to soak’ | OJ *nure*-  ‘to get wet’  J *nur*- ‘paint’  < pJ **nura*- |  |  | MMo. *nur*-  'to be wet, soak'  < pMo **nor*- |  |
| pA **deb*-  ‘to soak’ |  |  | Evenki *dewe*- 'to dye, paint’  < pTg **debe*- | WMo. *debte*-  'to become wet, soak'  WMo. *debel* ‘clothes’  < pMo **debe-* | Tatar *ǯebe*-  'to become wet, soak'  < pTk **yẹbi*- |
| pA **olo*  ‘hemp’ |  |  | Ma. *olo*  ‘hemp’  < **olo* | MMo. *olosun*  ‘hemp’  < pMo **olo-sun* |  |
| pJK **asam*  ‘hemp’ | OJ *asa*  ‘hemp’  < pJ **asa* | MK *·sam* ‘hemp'  < pK **ʌsam* |  |  |  |
| pJK **parʌ-*  ‘to sew’ | OJ *pari*  ‘needle’  < pJ **paru-i* | LMK *palol* ‘needle’  < pK **palʌ-l* |  |  |  |
| pJK **pata*-  'to weave (cloth) with a loom’ | OJ *pata*  'loom, woven cloth'  < **pata*- | MK ·*pca*-  'to weave’  < **pʌcʌ*- |  |  |  |
| pJK **pu*- 'to spin, twist (thread)’ | OJ *pu* '(woven) stitch’  < **pu* | MK *pwuk* ‘shuttle (of loom)’  < **pu*-*k* |  |  |  |
| pJK **kama*-  ‘to wash, soak’ | OJ *kamu pata* ‘multi-colored woven cloth’  OJ *kam*-  ‘to brew’  < pJ **kamǝ*- | MK ·*kom*-  'to bathe, wash'  pK **kʌmʌ-* |  |  |  |

**Detailed historical comparative linguistic dataset**

**of Transeurasian cognates underlying our analysis in Table S1**

**(1) pTEA **nap*- ‘to make rope’**

**Japonic**: pJ **nap*- ‘to make rope’ (pJ *-*a* deverbal nominalizer; Sakakura 1966: 286–303, Robbeets 2015: 156)

J *nau* (B), OJ *nap*- ‘twist, plait, weave (into rope)’, J *nawa* (2.3), OJ *napa* 'rope'

**Koreanic**: pK **nap*- ‘twist, spin’

K *nah*- ‘spin, weave, make yarn’, K *kkunapwul* ‘ a string of cord’ < *kkun* ‘cord, string’ + **nap*- ‘twist, twine, spin’ + -*wul* deverbal nominalizer, Kyeylim Yusa phonogram EMK *na(h)* ‘string’

**Tungusic**: pTg **nap*- ‘to make rope’ (pTg *-*ki* resultative nominalizer; Robbeets 2015: 407)

Ulcha *lāxị*, Orok *lāpụ*, Na. *lāpị*, Oroch *lappi* ‘tiers, straps (for skis)’

The Tungusic words for ‘tiers, straps (for skis)’ can be derived with the resultative deverbal noun suffix pTg *-*ki* from an underlying verb **nap*- ‘to make rope’. Proto-Tungusic lacks initial liquids, except **l*- going back to original nasal **n*- assimilation before labial consonants (Poppe 1960: 74, Robbeets 2005: 69).

Twining can produce cloth, string or rope. Cords for making traps and nets have been found in a number of upper Paleolithic sites across the world (Tedlock 2009: 66, Soffer et al. 2000: 512-514). Therefore, making rope is generally older than weaving textiles.

**(2) pTEA **nup*- ‘to sew'**

**Japonic**: pJ **nup*- ‘to sew, stitch’

J *nuu* B*,* OJ *nup*- ‘to sew, stitch, embroider’, Yoron (Amami) *nuuju*ɴ 'to sew', Shuri (Okinawa) *nooju*ɴ, Hirara (Miyako) *nuu* 'to sew', Igarashi (Yaeyama) *nooŋ* 'to sew', Yonaguni *nuŋ* 'to sew', pR **noCu*- 'to sew',

**Koreanic**: pK **nupi*- ‘to sew, quilt’

MK *nwu(·)pi*- ‘to quilt’, MK *nwu·pi* ‘quilting’

**Tungusic:** pTg **nup*- ‘to prick, pierce’: Evk. *lupa*- ‘to prick’, *lupu:-* ‘to go through, pierce’, Even *nụbas an*- ‘to prick’, Neg. *lepu*- ‘to pierce’, Na. *loqpa*- ‘to prick (intr.)’, Olch. *loqpa*- ‘to prick oneself’, *loqpụ(n)* ‘a splinter’, Orok *lụkka*- ~ *lụqpa*- ~ *lupqa*- ‘to prick oneself, to impale oneself upon smth; to prick smth’

The Tungusic verb stem is probably a compound of pTg **nup*- ‘to prick, pierce’ with a suffix *-*kA*-, perhaps the alternant of the inchoative suffix pTg *-*xA*- in voiceless clusters (see Robbeets 2015: 259.) Poppe (1960, 74) finds that the initial *l*- in the Tungusic languages is a secondary development from an original **n*-: “Das anlautende *l* im Mandschu-Tungusischen ist sekundärer Herkunft und geht gewöhnlich auf ein anlautendes **n* (meistens vor einem folgenden **m*) zurück.” This view is consistent with the general absence of initial liquid phonemes across the Transeurasian languages. The environment in which this development takes place needs further study, but it should probably be extended to the position before *-*PK*- clusters, e.g. pTg **nabga:n*- ‘to glue, stick’ in Evk. *labgan*-, Even *nabgan*-, Neg. *labga:n*-, Orok *lamba*-, Ud. *lagbamu*-; pTg **nobgi* ‘squirrel nest’: Evk. *lopi* (dial. loki:); Neg. *lo:bị*, Ulcha *logbụ*, Na. *lo:bị*, Ud. *loi;* pTg **napki* ‘tiers, straps (for skis)’: Ulcha: *la:xị*, Orok *la:pụ*, Na. *la:pị*, Oroch *lappi*; pTg **napku*- ‘to insert, hang: Evk. *lapku*-, Even *napkü*-. Note that Even consistently retains the initial nasal here. Sewing enters the archaeological record with leather clothing, and is generally older than weaving textiles.

**(3) pTEA **sili*- 'to sew’**

**Koreanic**: pK **sili* 'thread' < ? pK **sil(i)-* 'to sew, tie together'? (pK *-*i* deverbal noun; Robbeets 2015: 459)

K *sil*, MK ̈ *sil* ‘thread’

**Tungusic**: pTg **sira*- 'to sew together, tie together'

Evk. *sira*- ~ *hira*- ‘to piece down, to lengthen, to add (cloth, belt)’, Even *hịraq*- ‘to piece down, to lengthen, to add (a belt, a rope), to connect, to add’, *hịraqan* ‘phalanx of a finger’, Neg. *seya*- ~ *siya*- ‘to lengthen, continue a belt, a rope; connect two parts’; *siya:n* ~ *siyə:n* ‘thread; way (figurative)’; *siyəktə* ‘thread’; Orok *sịra*- ‘to lengthen, to piece down; to add a belt, a rope etc.; to connect together ends of a rope, a thread etc.’; Ma. *sira*- ‘to continue, follow; to connect, tie together; to inherit’, *sirame* ‘next (in sequence); step- (e.g. *sirame ama* ‘stepfather’; *siran* ‘continuation, succession, sequence, order’; Sibe *sira*- ‘to inherit; connect; to join; to continue’; Jur. *sir(a)-ru* 'inherit'; Olcha *sịra*- ‘to connect’, *sịra-ǯụ*- ‘to add; to marry a widow of the elder’s brother; to inherit’, *sịra-čụ*-*ǯụ*- ‘to tie together’; Na. *sera*- ‘to sew together cords, threads; to continue telling a fairy-tale which was interrupted’, *sera-go*- ‘marry a widow of one’s elder brother; keep on fire of one’s clan; to tie together two cords’, *sera-kta*- ‘to tie together several cords, threads’, *sera-ča* ‘knot (to lengthen a cord, a thread); a cord, a thread (for lengthening)’; Oroch *siya*- 'to sew together, piece down'; Ud. *sǣ*-, *sǣ-si*- ‘to piece down, to lengthen (cloths), to sew together (climbing skins); to add (a rope, a belt)’; *sǣ* ‘cross joint of climbing skins (in piecing down)’

**Mongolic**: pMo **siri*- 'to sew, stitch, quilt'

WMo. *siri*- '1 to quilt, stitch (tr.)', Khal. *šire*- 'to make firm by sewing, to quilt', Bur. *šere*- 'to stitch (usually the sole of a shoe)', Kalm. *šir*- '1', Ordos *šire*-, *širi*- '1', Dgx. *šïri*- '1', Dag. *širi*-, *šire*- '1', Eastern Yugur *širǝ*- '1', Mgr. *śiri*-, *śirǝ*- 'to cover a bed with a counterpane'

**Turkic**: pTk **sïrï*- 'to sew, stitch, quilt'

Karakhanid *sïrï*- 'to sew, to stitch, to quilt or smock a garment', Tk. *sïrï*- 'to sew tightly, quilt', Az. *sïrï-* 'to quilt; to foist, impose', Tkm. *sïra-* 'to sew, to stitch, to quilt', Tat. *sïr*- 'to quilt', Kaz. *sïr-* 'to stitch', Nog. *sïrï-* 'to quilt', Bash. *hïr-* 'to quilt', KKalp. *sïrï-* 'to sew across; to quilt', Kum. *sïrï-* 'to sew; to quilt; to fasten, attach'*,* KBalk*.* sïrï- ‘to quilt’, Uig. *širi-* 'to quilt', Khak. *sïrï-* 'to sew; to quilt', Tuva *sïrï-* 'to sew; to quilt'

Given the rising tone, MK ̈ *sil* ‘thread’ can be derived from an original polysyllabic form, whereby the contraction was likely to be due to the equality of the vowel in both syllables, thus pK **sili* 'thread'. Given the reconstruction of a deverbal noun noun suffix pK *-*i* ~ *ø*, attested in e.g., MK *hal*- 'to slander' → MK *hali* 'slandering' and MK *nwu·pi*- 'to quilt' → *nwu·pi* 'quilting', it is possible that the Korean form originally derived from a verb pK **sil(i)-* 'to sew, tie together'. Note that the deverbal derivation from 'to sew, tie together' as 'thread' is also attested in the Tungusic languages, e.g. Na. *sera*- ‘to sew together (cords, threads)' → *sera-ča* ‘cord, thread'.

**(4) pTEA **pɔrɔ*- ‘to weave (cloth)’**

**Japonic**: pJ **orə*- ‘to weave’

J *oru* A ‘weave’, OJ *oro_2_s*- ‘deign to weave’, Asama (Amami) *Ɂujuɴ* 'to weave', Shuri (Okinawa) *Ɂujuɴ* 'to weave', Irabu (Miyako) *uï* 'to weave', Ishigaki (Yaeyama) *uruɴ* 'to weave', Yonaguni *úrun* 'to weave'

**Koreanic:** pK **olʌ* ‘unit of woven fibers, component of woven fabric’

K *o:l*, MK ¨*wol* ‘strand of rope, ply, warp’, K *olk*- ‘to tie up, bind, weave’ (< pK **olʌ* ‘woven fabric’ + ·*kʌ*- inchoative; Robbeets 2015: 258)

**Tungusic**: pTg **poro*- ‘to spin (nettle and hemp threads); to rotate, turn’: Evk. *horol*- ‘to spin, whirl, go around’, *horoli:* ‘around’, Neg. *xoyil*- ~ *xoyol*- ‘to spin; to circle; to eddy (about water)’, *xoyil* ~ *xoyol*, *xoxsin* ‘eddy’, Ud. *xoli*- ‘to circle (about birds); to circle, to whirl’, Sibe *foro*- ‘to spin; to turn’, Ma. *foro*- ‘to spin; to turn, to turn around, to face, to turn toward’, *forko* ‘spinning wheel’, *foron* ‘swirl, curl, whirl; rotation (of an arrow between the fingers)’, *foro-no*- ‘to turn (in that direction)’, *forontu* ‘curly, having curly hair’, Olcha *pori*- ‘to weave (nets)’, *porpu(n*) ‘a spindle; a device for weaving nets’; Oroch *porpụ* ‘a spindle for spinning nettle and hemp threads’

**Mongolic**: pMo **poro*- ‘to tie around, entwine; rotate, turn’ in **poro-go*- ‘to wrap’ (*-*gA*- causative) and **poro-ti*- ‘roll, rotate’ (*-*ti*- intensive)

WMo. *oriya*- ‘1 to tie around, entwine, wrap, bandage, wind, roll (tr.)’, *oruɣa*- ‘1’, *orči*- ‘2 to turn around, roll, rotate’ (intr.)’, MMo. *hura*- ‘1’, *xorči*-, *horči*-, *orči*- ‘2’, *orčul*- ‘2’, Khalkha *orō*- ‘1’, *orči*- ‘2’, Buriat *oŕo:*- ‘1’, *oršo*- ‘2’, Kalmuck *ora:*- ‘1’, *orčǝ*- ‘2’, Ordos: *oro:*- ‘1’, *orčin* ‘around’, Dgx. *xoro*- ‘1’, Baoan *horǝ*-, Dagur *oŕe:*-, Eastern Yughur *horo:*-, Mgr *furo:*-, *xuro:*- ‘1’

**Turkic**: pTk **pö:r*- ‘to plait, weave’: OT (Karakh.) *ör*- ‘to plait (hair or other fibers)’, MTk *ör*- ‘1 to weave, plait, twist things together’, *örmek* ‘cloth woven from camel hair’, Kirg. *ör*- ‘1’, Kaz. *ör*- ‘1’, *örĭm* 'woven part of sth (e.g. of a whip); bundle', Nog. *ör*- ‘1’, Bash. *ür*- ‘1’, Karaim *ör*- ‘1; to spin’, Kkp. *ör*- ‘1’, Tat. *ör*- ‘to plait, to knit, to darn, to interlace, to interweave, to build (a wall), to lay bricks or stones in a building’, Tk. *ör*- ‘1’, Az. *hör*- ‘1; to knit’, Tkm. *ö:r*- ‘1’, Gag. *yör*- ‘1; to knit’, Uz. *ọr*- ‘1’, Uig. *ö(r)-* ‘1’*,* Yak. *ör*- ‘1’, *örǖ* ‘plaiting’, Dolg. *ör*- ‘to plait, bind together, wind’, *örǖ* ‘plaiting’, Khalaj *hiri*-, *hör*- ‘to plait’, Chu. *var* ‘best part; sort of fiber; flax', *vĕren* ‘cord, rope’

The expected reflex of pTEA **p*- is **p*- in proto-Japonic and proto-Koreanic However, an initial labial stop sporadically drops before a (long?) rounded pJK **o(:)*, as it probably also did in the reflexes of pTEA **bɔ:l*- ‘to sit down, become, be’ in Japanese (pJ **wo*- ‘to sit, be’ in OJ *wi*- ‘to sit, be’, OJ *wor*- ‘to be, exist’) and Korean (pK **o*- ‘to be’ in MK *-^.^w^u^/o-* modulator). Old Japanese makes no distinction between *o_1_* (< **o*) and *o_2_* (< **ə* ) in initial position, but I have opted for **o* in pJ **orə*- ‘to weave’ because it entails a regular external correspondence. The root-final vowel of pJ **orə-* is an irregular fit, which may be due to vowel reduction in root-final position.

The regular reflexes of pTg **p*- are Nanai/ Olcha/ Orok *p*-, Manchu *f*-, Evenki/ Even *h*-, Negidal/Oroch/ Udehe *x*- and Solon Ø (Benzing 1956: 981). Except for Oroch *po:rpu*, *po:rfu* ‘spindle’, which is probably a borrowing from Olcha, the cognates are thus corresponding regularly and suggesting the reconstruction of an initial pTg **p*-.

The initial labial stop pMo **p*- is regularly preserved in the peripheral Mongolic languages, notably as *f*- in Monguor *furō*-, as *h*- in Shira-Yughur *horō*- or Baoan *horǝ*- and as *x*- in Dongxiang *xoro*-, but it disappeared in the central Mongolic languages.

For Turkic, it is commonly assumed that word initial pTk **p*- developed over a bilabial fricative into *h*-, leaving only a trace in Khalaj *h*- and finally disappeared in most of the contemporary Turkic languages. Given the attestation of Khalaj *hör*- ‘plait’ it is legitimate to reconstruct pTk **pö:r*- ‘to plait, weave’. The long vowel in Proto-Turkic is expected to yield Chuvash **varen, while the actual Chuvash reflex is *vĕren* ‘cord, rope’. Savelyev (pc.) suggests that the deverbal noun pTk **örken* ‘rope’, having cognates in some other Turkic languages apart from Chuvash, is derived from pTK **ö:r*- ‘to plait’, with shortening of the vowel before a consonant cluster.

The semantic association between 'to weave' and 'to spin' can be observed in the polysemy between Karaim *ör*- ‘to weave, plait; spin’ (Baskakov et al. 1974: 442) or in Oroch *tompo*- ‘to spin threads; to weave a net’ below, examples in which an original meaning 'to weave' developed into 'to spin'. This is also the direction of the semantic shift assumed for the Tungusic and Mongolic proto-forms.

Weaving is labor-intensive and technologically complex, requiring a loom system. Only a society with food-surplus can invest in the technology and labor required (Barber 1995). Therefore, weaving is generally linked to agriculture.

**(5) pTEA **tɔmʊ*- 'to spin’**

**Japonic**: pJ **tumu* 'spindle'

J *tumu* (2.4), OJ *tumu* 'spindle', J *tumug*- (B), OJ *tumug*- ‘to spin, make into yarn’, Shuri (Okinawa) *çing*- (B) 'to spin'

**Tungusic**: pTg **tom(u)-* > *tumu*- 'to spin' (pTg *-*ku* ~ *-*ko* deverbal instrumental noun suffix, e.g. Na. *xado*- 'to mow' → *xadoko* 'scythe')

Even *tum*- '1 to spin, wind, coil, spool, wrap', *tumenŋe* 'thread wind around a bobbin', *tomqo*- ‘to spin strings for threads; to spin threads’, *tomqon* ‘spinning of strings; yarn’; Evk. *tum*- ' 1', *tomko* ‘a thread’; *tomko*- ‘to spin threads; to tie with a thread’; Neg. *tum*- '1', *tumu* 'string of thread, parcel, roll', *tomko* ‘thread’, *tomko*- ‘to spin threads’; Solon *tum*- 'to bind, knit, string together, tie', *toŋxo*- ‘to spin threads’; Oroch *tumu*- '1', *tompo* ‘a sinew or nettle thread’, *tompo*- ‘to spin threads; to weave a net’; Olcha *tumu*- '1', *toŋpo* ‘short threads of nettle or hemp’, *toŋpo*- ‘to spin threads’; Orok *tumu*- '1', *toqpo*- ~ *topqo*- ‘to spin threads, rope, cord’; *toqpo* ~ *topqo* ‘thread; rope’; Na. *tumu*- '1', *tompo*- ‘to spin threads of fishskin’, *tompo* ‘threads made of fishskin’; Ud. *tompo*- ‘to spin threads, ropes’

**Mongolic**: pMo **tomu*- ~ **tamu*- 'to spin'

WMo. *tomu*-, *tamu*- '1 to twist or spin thread or rope', MMo. *tamu*- ~ *toma*- ~ *tomu*- ~ *doma*- '1', Khal. *tam*- ~ *tom*- '1', Bur. *tomo*- '1', Ordos *tamu*- '1', Kalm. *tom*- ~ *töm*- 'to twist, twine; to string together (rope), make rope (by turning horse hair between the hands)', Eastern Yugur *tomu*- ~ *tɔmɔ*- ~ *tomə*- '1', Dgx. *tomu*- '1', Bao. *tomǝl*- '1', Mgr. *tomu*- ~ *tamu*- '1'

The Tungusic reconstruction displays a vowel alternation between **tom(u)-* and **tumu*- 'to spin'. Given the preservation of **tom(u)-* in derived nouns with the instrumental suffix and re-verbalizations thereof, I assume that **tom(u)-* assimilated to **tumu*-.

In Mongolic we find a vowel alternation between **tomu*- ~ **tamu*- 'to spin', reminiscent of the alternation between pMo **dolaan* 'seven' and **dalan* 'seventy' (Nugteren 2011: 512).

**(6) pTEA **giri*- 'to cut (cloth)'**

**Japonic**: pJ **kira*- ‘to cut (e.g. cloth)’

J *kir-* B*,* OJ *ki_1_r-* ‘to cut, shear, chop’, OJ *ki_1_ras-* B ‘to run/ sell out of’, J *kireru* B*,* OJ *ki_1_re-* ‘to be sharp, get cut, run out’, J *kiri* 'texture', J *kiri*, OJ *ki_1_ri* 'awl, pointed tool for piercing small holes (as in cloth or leather)', Yamatohama (Amami) *kiruri* 'to cut', Shuri (Okinawa) *ciju*ɴ 'to cut', Irabu (Miyako) *cïï* 'to cut', Ishigaki (Yaeyama) *kïsuɴ* 'to cut', Yonaguni *ccùn* 'to cut', pR **kiri*- 'to cut' (Thorpe 1983: 276)

**Tungusic:** pTg **giri*- ‘to cut out (e.g. cloth, paper, pelt)’: Evk. *gir*- ~ *ger*- ~ *kir*- ‘to cut out (with scissors)’, *giri-ptun* ‘cloth piece’, Even *gịr*- ‘to cut out’, Neg. *gey*- ~ *giy*- ‘to cut out’, Ma. *giri*- ‘to trim with a knife or scissors, to cut evenly, to cut a strip’; *giri-n* ‘strip; section, area’; *gir*-*dan* ‘cloth or strips of pelts cut with scissors; evenly cut slices of meat; pennant; border trim on a banner’; *giriku*: ‘a small knife for trimming skin, paper, and cloth’, Olcha *gịrị*- ‘to cut out’, *gịrsụ* ‘woman’s knife for cutting things out’, Orok *gịrị*- ‘to cut out’, *gịrị-ptụla* ‘cloth pieces, remnants’, Na. *gere*- ‘to cut out with a knife’, *gerego*- ‘to recut out, to cut out anew’, *gerekta*- ‘to cut out many objects’, *gerekto* ‘cut out’, *geremsa*, *gereptola* ‘cloth rags’, *gereče*- ~ *gerenase*- ‘to cut out many times’, *gerso:* ‘woman’s knife for cutting objects out’, Oroch *gi:-* ~ *giyi*- ‘to cut out with a knife’, Ud. *gi:*- ~ *gi:na*- ‘to cut out with scissors’

**Turkic:** pTk **kïr*- ‘to cut strips of hair, pelt or cloth, scrape, shave’: OT (Karakh.) *kïr*- ‘to scrape, strip (hair), pluck out (hair), to cut off’, Tk. *kïr-* 'to break, split (wood); to rough-grind, crush (grain); to hurt, injure', Az. *ġïr-* 'to break; cut', Tkm. *ġïr-*'to scrape', Gag. *ḳïr-* 'to break, crack', Tat. *ḳïr-* 'to break; to scrape; to shave; to grate; to whet; chafe', Khak. *xïr*- ‘to cut; to scrape; to shave; to chafe', Kirg. *kïr*- 'to scrape; to shave', Kaz. *kïr*- 'to scrape; to shave; to chafe', Nog. *kïr*- 'to scrape; to shave', Bash. *kïr*-''to scrape; to shave; to grate; to chafe; to whet', Karaim *kïr*- 'to scrape; to shave', Kkalp. *kïr*- 'to scrape; to shave', Uz. *kir*- 'to scrape; to shave; to break', Uig. *ki(r)*- 'to scrape; to shave; to break', Yak. *kïrïy-* ‘to cut’, *kïra* 'small', Dolgan *kïrïy-, kïrpala:-* 'to cut', Tuva *kïr*- ‘to break; to exterminate all without exception', Khalaj *kïr*- ‘to break’, Chu. *xïr*- 'to scrape'

The Senchū Wamyō Ruijūshō (AD 930), an Early Middle Japanese lexicon, among others covering vocabulary for textile, employs the verb MJ *kir*- 'to cut' as the standard verb for cutting in the production process of textile (Omura & Kizawa 2017: 455). The semantic context of cloth production is further supported by deverbal nouns meaning 'texture' or 'awl'. Simularly, given the deverbal nouns meaning 'strip of cloth or pelt', the Tungusic verb is specialized for the cutting of cloth, paper or pelt. The denominator that is most widespread across all Turkic languages is ‘to shave, to scrape (leather with a knife)’, while some Turkic languages saw the development of secondary meanings such as ‘to shave oneself’, ‘to cut hair’, ‘to break’. Scraping means ‘cutting off or removing by repeated strokes of an edged instrument’, while shaving means ‘cutting off hair, beard or pelt in thin layers close to the skin’. This observation leads us to reconstruct ‘to cut strips of hair, pelt or cloth’ as the Proto-Turkic meaning. Therefore, the common semantic denominator across the Transeurasian languages seems to be the cutting of strips of cloth and the verb may have its origin within a context of textile production.

The Mongolic forms WMo. *kira*-, *kiru*-, Khal. *x´ar*- and Kalm. *kur*- that support the reconstruction of pMo **kira*- ‘to cut into small pieces, mince’ have been omitted since they can be assumed to have been copied from Turkic. If they were cognates, we would expect an initial voiced velar (**g*-) in Mongolic. The borrowing of this form with the meaning of ‘to cut into small pieces’, supports our semantic reconstruction in Proto-Turkic.

The Chuvash verb *xïr*- 'to scrape' has an irregular vowel correspondence and may thus be due to borrowing. If it is indeed borrowed, the most plausible model languages would be Middle Kipchak or Tatar. However, we would expect the shape Chuvash ***xăr*- for a loan from Middle Kipchak *kïr*- and ***kïr*- if it were borrowed from the contemporary Tatar verb *kïr*-. Therefore, Savelyev (pc) suggests that PTk **kạŕ*- ‘to dig’- and **kïr*- ‘to scrape’ have contaminated to yield Chuvash *xïr*- ‘to scrape’, reflecting the form of the former, but the meaning of the latter word.

**(7) pTEA **simi*- ‘to soak’**

**Japonic:** pJ **sima*- ‘to soak, permeate’

J *sime*- ‘to be damp, soaked’, J *simi*- 'to soak', *simi* 'stain', OJ *sim*- ‘to pierce, soak, sink, permeate’, Shuri *suum*- ‘to soak’

**Mongolic:** pMo **sime*- ‘to soak, moisten, suck up’

WMo. *sime*- ~ *simi*- ‘1 to draw a liquid into the mouth, suck up, sip (tr.)’, *simed*- ~ *simede*- ‘to become soaked with, become saturated, become absorbed (intr.); to penetrate’, *simedke*- ‘to soak in, absorb (tr.)’, *sime* ‘3 essence, extract, sap, nourishment’, *simejin ariki* ‘liquor made from fermented milk’, MMo. *šimi*- ‘to soak; to suck’, Khal. *šime*- ‘to suck’, *šimte*- ‘to become soaked’, Khal *šime*- ‘1’, *šimde*- ‘2’, *šim* ‘3’, Buriat *šeme*- ‘2’ *šeme* ‘3’, Kalm. *šimǝ*- ‘to suck, suck out’, *süm*- ‘to suck (of cattle)’, *šime:ldǝ*- ‘to become absorbed, to penetrate’ (< **sime-ge-ldü*-), *šim* ‘juice, taste, nutritious liquid’, *mali:n šimǝ* ‘edible products, the juices of cattle (i.e. butter, cheese, milk, fat)’, Ordos *šime*- ‘2’, *šime* ‘3’, Dagur *šime*- ‘2’, *šim* ‘3’, Eastern Yugur *šǝme*- ‘2’, *šǝme* ‘3’, Mgr. *šǝme*-, *šǝmu*- ‘2’, *śime*, Mogh. *šimi* - 'to suck', Dgx. *ʂïməi*- ‘to sip, take in the mouth and taste’

Although the Korean verb K *sumi*-, MK *·su·mui*-‘to soak, permeate, infiltrate, sink into’ is a good semantic match and looks similar, the Korean vowel -*u*- < pK **ɨ* represents an irregular sound correspondence.

A plausible cognate in Tungusic is Manchu *sime*- ‘to soak, to moisten, to seep into; to favor’, *simi*- ‘to suck’ and *simen* ‘moisture, juice, secretion, nutritive fluid’. However, the poor distribution of this verb in Tungusic, restricted to Manchu, signals that this word may be borrowed from Mongolic. If this is indeed the case, the borrowing yields additional support for reconstructing the Proto-Mongolic meaning as ‘to soak, to moisten’, preserved in the passive derivative pMo **sime-de*- ‘to become soaked’ and the derived noun **sime-n* ‘moisture, liquid’

In Turkic, we can reconstruct pTk **simü-r*- ‘to suck’ on the basis of Karakhanid *simür*- ‘1 to suck, swallow’, Turkish *süm*-, *sümür*- ‘1’, Az. *sümür*- ‘1’, Tkm. *sümür*- ‘1’, Kaz. *simir*- ‘1’, Nog. *simir*- ‘1’, Bash. *hĭmĭr*- ‘1’, Kkp. *simir*- ‘1’, Uz. *simir*- ‘1’ and Uig. *sümür*- ‘1’. The stem-final -*r-* in Turkic may be identified as the causative suffix -*(U)r-*, which has developed secondary non-valence changing meanings such as intensive and iterative. In addition to Turkish *süm*- ‘to suck’, other froms such as Yak. *sim*- ‘to stuff’ and Cuman *sim*- ‘to swallow’ seem to reflect the underived verb root (Servotyan 1974-2003 vol. 7: 378–379). That the latter two verbs have an irregular vowel correspondence may be due to the descriptive nature of the root. However, the limited distribution of the Turkic verb, and the observation that Turkic only preserves the secondary meaning ‘to suck’ makes us suspect that this form may be borrowed from Mongolic.

**(8) pTEA **ulu*- ‘to soak, wet’**

**Japonic:** pJ **uru*- ‘to be wet’ (pJ *-*pa*- ~ -*pə*- reflexive-anticausative; Robbeets 2015: 292-294)

OJ *urup*- (?B) ‘to get muddy, be wet’, J *uruow*-, OJ *urupop*- (?B) ‘to get damp, get moist, receive profits, get enriched’, OJ *urum*- (?B) ‘to get wet, moist’

**Koreanic:** pK **uli*- ‘to soak’

K *wuli*-, MK *wuli*- ‘to steep, soak, bleach’

**Tungusic:** pTg **ula*- ‘to wet, soak’ (pTg *-*p*- anticausative; Robbeets 2015: 296-298)

Evk. *ula*- ‘to soak, to wet (legs); to become wet; to melt (about falling water snow)’, *ulap*- ‘to become wet’ (Evk. -*p*- anticausative; Robbeets 2015: 298); Even *ụl*- ‘to soak, to wet’, *ụlab*- ~ *ụlap*- ‘to get wet’, Sol. *ụlakku:* ‘wet’, Ma. *ulga*- ~ *ulha*- ‘to wet, dampen, dip in a liquid’, *ulgaku:* ‘inkwell, well for ink on an inkstone’, Jur. *ul(h)a*- ‘to wet, to dampen, to dip in liquid’, Orok *ụla*- ‘to soak; to wet’, Na. *ụlarïko:* (dial.) 'wet', Ud. *ula*- ‘to soak, to wet; to set a net’, *ula-sa*- ‘to become wet (about dried fish); to be settled (about net)’

The semantic association between 'be wet' and 'to soak' is legitimate given the polysemy attested in Even *ula*- ‘to soak, to wet (legs); to become wet'.

**(9) pTEA **nɔr*- ‘to soak, be(come) wet’**

**Japonic**: pJ **nura*- ‘to be(come) wet’

J *nure*-, OJ *nure*- (A) ‘to be(come) wet, damp; to come undone, loose’, Yoron (Amami) *nidituɴ* 'to be wet', Shuri (Okinawa) *ndir*- (A) 'to get wet', *ɴditooɴ* 'to be wet', Hatoma (Yaeyama) *zoori bee* 'to be wet'

**Mongolic:** pMo **nor*- 'to be(come) wet, soaked'

WMo. *nor*- 'to become wet, soaked, drenched, damp, moist (intr.)', *norɣa-* 'to wet, moisten, soak (tr.)', MMo. *nur*- 'to be wet, soak', *norma* 'moistened', Khal. *nor*- 'to get wet', Bur. *noro*- '1 to become wet, soaked', Kalm. *nor*- 'to become wet', *norɣa:-* 'to make wet', *norɣu:(n), noru:* 'wet', Ordos *nor*- '1', Dgx. *noro*- ~ *nuru*- ~ *noru*- '1', Dag. *noir*- '1', *noirga*:- 'to make wet', Mgr. *no:ri*- ~ *nori*- '1'

Since ‘to soak’ means to ‘be(come) immersed in liquid (such as water)’, it is semantically overlapping with ‘be(come) wet’ and both meanings can be reconstructed to the Transeurasian proto-language.

**(10) pA **deb*- ‘to soak’**

**Tungusic:** pTg **debe*- 'to saturate with liquid, impregnate with dye’

Evenki *dewe*- 'to dye, paint', Even *dewe*- 'to dye, paint', Negidal *dewekse* 'paint', Manchu *debe*- ‘to overflow, run over, to flood’, Ulcha *dewekse* 'paint', Orok *dewe:*- 'to dye, paint', Oroch *dewukse*, *dewekse* 'paint'

**Mongolic:** pMo **debe*- 'to soak (e.g. clothes), make wet'

WMo. *debte*- ~ *debtü*- ‘1 to be soaked, steeped, saturated with a liquid; to swell, expand (intr.)’ (pMo *-*dA* passive), *debtege*- ‘to soak or steep in a liquid, dampen, moisten, soften’, e.g. *amu debtege*- ‘to soak grain’, WMo. *debül*- ‘to spout, gush, boil, bubble, to overflow’ (pMo. *-*l*- iterative), MMo. *debul*- 'to boil', WMo. *debege* ‘marshy place, damp or wet area, alpine grassland’ (pMo *-*GA* place suffix), WMo. *debel* ‘clothes; long garment, dress, gown, robe’ (pMo *-*l* deverbal noun, e.g. WMo *ükü*- ‘to die’ -> *ükül* ‘death’), Khalkha *devte*- ‘1’, Buriat *debte*- ‘1’, Kalmuck *deptǝ*- ‘1’, Ordos *debte*- ‘1’, Dagur *debte*-, *derte*- ‘1’, Eastern Yogur *debte:-* ‘1’, Monguor *tǝbdē*-, *tudē*- ‘1’, Mogol *debtäl*- `to make fall into the water'

**Turkic:** pTk **yẹbi*- 'to become wet, soak'

Karakhanid *yebe* 'dampness', Tatar  *ǯebe*- '1 to become wet, soak', Uzbek *ivi*- '1’, Uig. *ivi*- '1, Shor  *čibi*- '1, Oirat *d́ibi*- '1’, Chuvash  *śǝʷve* 'whey', Yakut *sibi:n*- 'fresh', Kirghiz *ǯibi*- '1’, Kazakh  *žibi*- '1’, Noghai  *yibi*- '1’, Bashkir *yebe*- '1’, Karaim *yibi*- '1’, *ibi*- '1’, Karakalpak  *žibi*- '1’, Kumyk  *yibi*- '1’

The Mongolic verbs display a semantic development from ‘to soak, immerse into liquid’ over ‘to be soaked, be saturated with liquid’ to ‘swell, expand, overflow’, i.e. ‘be over-saturated with liquid’. The latter meaning is attested in the Manchu verb *debe*- as well, while the other Tungusic verbs mean 'to dye, paint'. The common semantic denominator in Tungusic therefore seems to be ‘to saturate or impregnate with liquid such as water or dye’. This makes the comparison with the Mongolic and Turkic verbs meaning ‘to soak’ acceptable and leads to the reconstruction ‘to soak’ as the meaning of the proto-Altaic form.

**(11) pA **olo* ‘hemp’?**

**Mongolic:** pMo **olo* ‘hemp’ (pMo *-*sun* collective),

MMo. *olosun* ‘hemp’*,* WMo. *olosun*, Khal. *ols(on)*, Kalm. *olsn*, Dag. *olso,* Ord *ulusu*, Bur *ulha(n),* Eastern Yog. *losən*, Mgr. *losə*

**Tungusic:** pTg **olo* ‘hemp’?

Manchu *olo* ‘hemp’

The evidence for the original vocalism in proto-Mongolic **olo-sun* is ambiguous: Eastern Yoghur *losən* and Monguor *losə* support the reconstruction of **ola-sun* instead, while Ordos

*ulusu* and Buriat Bur *ulha(n)* point to **ulu-sun*, although the Ordos form can also go back to **olu-sun* (Nugteren 2011*)*.

The reconstruction of the word for ‘hemp’ in proto-Tungusic is speculative as it only occurs in Manchu. Therefore, borrowing from Mongolic into Manchu may be a more parsimonious explanation.

**(12) pJK **asam* ‘hemp’?**

**Japonic**: pJ **asa* 'hemp'

J *asa* (2.3) ‘flax (plant), hemp (plant), ramie, jute, linen’, OJ *asa* ‘hemp’

**Koreanic**: pK **ʌsam*

K *sam*, MK *·sam* ‘hemp'

The Japanese term *asa* is a generic term referring to bast fibers such as hemp (*Cannabis sativa Linne*) and false nettle (various species of *Boehemia*), often called ramie or Chinese grass in English. Kitamura and Murata (1973) mention that the *Boehmeria nivea L. Gaud* was brought from China already in ancient times. OJ *so_1_* ‘hemp, cloth’ is probably not related with OJ *asa* ‘hemp’, but a possible cognate may be MK *swom* ‘cotton’ (Francis-Ratte 2016).

Whitman (1985: 232), Vovin (2010: 173) and Francis-Ratte (2016) suggest that the high tone of MK *·sam* ‘hemp' indicates the loss of an initial vowel in pK **ʌsam.* Francis-Ratte proposes that the final -*m* in the Korean form originates from a compound with the Sino-Korean loan morpheme *ma* (麻) 'hemp', e.g. in the Sino-Korean synonym *taema* (大麻）'hemp'. However, the Korean internal analysis remains speculative and the existence of an older Scythian form **sana* ‘hemp’ (cf. Old Indo-Aryan *śaṇ* ‘hemp’, Mayrhofer 1992–2001/2: 605; Gamkrelidze–Ivanov 1995: 570) may provide an alternative explanation for the Korean form as a *Wanderwort*.

**(13) pJK **parʌ-* ‘to sew’**

**Japonic**: pJ **paru-i* 'needle' (pJ *-*i* deverbal noun suffix; Robbeets 2015)

J *hari* (2.4), Iwate *haru*, OJ *pari*, EOJ *paru* 'needle', Shuri *haai* (B) 'needle'

**Koreanic**: PK **palʌ-l* (pK *-*l* deverbal noun suffix; Robbeets 2015)

MK *pa·nol*, LMK *palol* ‘needle’

In Middle Korean, *lVl* sequences are occasionally confused with *nVl* sequences, e.g. *anil soy* 'not being' is assimilated to *alil soy* ‘not being’. However, the change proposed here takes an opposite direction, assuming dissimilation from MK *palol* to *panol*, even if the attestation of MK *pa·nol* is older.

This etymology suggests that the nouns for ‘needle’ are independently derived in Japonica and Koreanic by way of separate deverbal noun suffixes, but that the underlying verb ‘to sew’ is cognate and goes back to a common form in proto-Japano-Koreanic.

**(14) pJK **paca*- 'to weave (cloth) with a loom’**

**Japonic**: pJ **pata* ‘loom, woven cloth’

J *hata*, OJ *pata* (2.2a) 'loom, woven cloth'

**Koreanic**: pK **pʌcʌ*- > **pcʌ*- 'to weave' (pK *-*i* deverbal nominalizer / adverbializer; *-*k* deverbal nominalizer, e.g. K *ilwu*- 'to achieve' → *ilwuk* 'achievement')

K *cca*-, MK ·*pca*- 'to weave, knit, tie up',

The polysemy between 'loom' and 'woven cloth' in the Japanese member of this etymology support the derivation of these nouns as instrumental and object nominalizations of an original verb 'to weave'. Note that the suffix -*a* is reconstructed as a deverbal noun suffix in proto-Japonic (Sakakura 1966: 286–303, Robbeets 2015: 156) .

In Manchu we find *fatan* 'the sole of a foot or a shoe; comb-like tool used for working silk on a loom, weaver's reed', but the technical term is probably a semantic extension of pTg **pata* 'sole, bottom, fundament'. A similar polysemy exists for Korean *patak* 'cloth, weave, texture' and *patak* 'bottom, sole'. MK *potoy*  'healds of a loom, series of wires attached to the loom frame' may be related here as well.

**(15) pJK **pu*- 'to spin, twist (thread)’**

**Japonic:** pJ **pu* 'twisted thread, weaving'

J *hu*, OJ *pu* '(woven) stitch, mesh, weave, knit, knot', J *ya-hu* (eight-weave) 'many stitches in a woven fence'

**Koreanic:** pK **pu*- 'to twist (thread)' (pK *-*i* deverbal nominalizer / adverbializer; *-*k* deverbal nominalizer, e.g. K *ilwu*- 'to achieve' → *ilwuk* 'achievement')

Ki *pi*-, MK *puy*- 'twisted (bound adverb), e.g. in K *pi:-thul*-, MK *puy-thul*- 'to twist, contort', *pi:-kko*:- 'to twist (thread), twist up', *pikki*- 'be bent, lie at an angle', *pi-kkule*-*may*- 'to tie, bind'; K *pwuk*, MK *pwuk* ‘shuttle (loom instrument)’ (< **pu-k* twist-NMLZ), Cheychwu *pi* 'shuttle' (< **pu-i* twist-NMLZ)

In Japanese, we also find J *hi*, OJ *pi_1_* 'shuttle', but the quality of the front vowel contradicts derivation from **pu-i* (twist-NMLZ) because that would lead to a vowel of the quality *i_2_* in Old Japanese.

**(16) pJK **kama*- ‘to wash, soak’**

**Japonic:** pJ **kamǝ*- ‘to chew, brew, dye’

OJ *kam*- ‘to brew’, J/OJ *kamos*- (B) ‘brew’, OJ *kamu-pata* ‘multi-colored woven cloth’, J *kamu* (B)*,* OJ *kam-* ‘to bite, gnaw, chew, masticate, eat’; Yamatohama (Amami) *xamuri* 'to eat', Asama (Amami) *kamyu*ɴ 'to eat', Shuri (Okinawa) *kamu*ɴ 'to eat', Hirara *kam* 'to bite', Ishigaki *kamuŋ* 'to bite', Yonaguni *kamuŋ* 'to bite'*,* pR **kamu*- 'to bite, eat'

**Koreanic:** pK **kʌmʌ-* 'to put in a bath'

MK ·*kom*-, K *ka:m*- 'to bathe, wash'

**Abbreviations**

Az. Azerbaijani

Bao. Bao'an

Bash. Bashkir

Bur. Buriat

Chu. Chuvash

Dag. Dagur

Dlg. Dolgan

Dgx. Dongxiang

Even Even

Evk. Evenki

Gag. Gagauz

J Japanese

Jur. Jurchen

Kalm. Kalmuk

Karakh. Karakhanide

KBalk. Karachay-Balkar

Krm. Karaim

Kkp. Karakalpak

Kaz. Kazakh

Khak. Khakas

Khal. Khalkha

Kir. Kirgiz

K Korean

Kum. Kumyk

Ma. Manchu

MK Middle Korean

MMo. Middle Mongolian

Mogh. Moghol

Mgr. Monguor

Na. Nanai

Neg. Negidal

Nog. Nogai

OJ Old Japanese

OT Old Turkic

Ord. Ordos

pJ proto-Japonic

pK proto-Koreanic

pMo proto-Mongolic

pTEA proto-Transeurasian

pTg proto-Tungusic

pTk proto-Turkic

Sol. Solon

Tat. Tatar

Tofa. Tofalar

Tk. Turkish

Tkm. Turkmen

Ud. Udehe

Uigh. Uighur

Uz. Uzbek

WMo. Written Mongolian

Yae. Yaeyama

Yak. Yakut

Yon. Yonaguni

**Additional references**

Barber, Elisabeth. 1995. *The Mummies of Urumchi*. New York: Norton.

Francis-Ratte, Alexander (2017). ‘Lexical recycling as a lens onto shared Japano-Koreanic agriculture’, in Martine Robbeets and Alexander Savelyev (eds), *Language Dispersal Beyond Farming*. Amsterdam: Benjamins, 75–92.

Nugteren, Hans. 2011. Mongolic phonology and the Qinghai-Gansu languages. Utrecht: LOT. Leiden University Ph.D. Dissertation.

Poppe, Nicholas 1960. *Vergleichende Grammatik der altaischen Sprachen. Teil 1, Vergleichende Lautlehre* [Porta Linguarum Orientalium, Neue Serie 4]. Wiesbaden: Otto Harrassowitz.

Robbeets, Martine. 2005. *Is Japanese related to Korean, Tungusic, Mongolic and Turkic?* (Turcologica 64). Wiesbaden: Harrassowitz.

Robbeets, Martine. 2015. Diachrony of verb morphology: Japanese and the Transeurasian languages. (Trends in Linguistics. Studies and Monographs 291.) Berlin: Mouton-De Gruyter.

Sakakura, Atsuyoshi. 1966. *Gokōsei no kenkyū* [Investigations in the composition of words]. Tokyo: Kadokawa shoten.

Sevortjan, Ervand V. (ed). 1974-2003. *Etimologičeskij slovar' tjurkskih jazykov*. [Etymological dictionary of the Turkic languages] Moscow: Nauka.

Soffer, O. J., Adovasio, M. & Hyland, D. C. 2000. The “Venus” figurines. Textiles, basketry, gender, and status in the Upper Paleolithic. *Current Anthropology* 41: 511–537.

Starostin, Sergej A., Anna Dybo & Oleg Mudrak. 2003. *Etymological dictionary of the Altaic languages*. Leiden: Brill.

Tedlock, Barbara. 2009. *The Woman in the Shaman's Body: Reclaiming the Feminine in Religion and Medicine*‬. London: Random House Publishing Group.

Thorpe, Maner Lawton. 1983. *Ryukyuan language history*. Los Angeles: University of Southern California Ph.D. dissertation.

Vovin, Alexander (2010). *Korea-Japonica: A Re-Evaluation of a Common Genetic Origin*. Honolulu: University of Hawai‘i Press.

Whitman, John Bradford. 1985. *The phonological basis for the comparison of Japanese and Korean*. Cambrige: Harvard University Ph.D. dissertation.
